# Investigating the Stirred Tank Bioreactor Co-Cultures of the Secondary Metabolite Producers *Streptomyces noursei* and *Penicillium rubens*

**DOI:** 10.3390/biom13121748

**Published:** 2023-12-05

**Authors:** Tomasz Boruta, Anna Ścigaczewska, Marcin Bizukojć

**Affiliations:** Department of Bioprocess Engineering, Faculty of Process and Environmental Engineering, Lodz University of Technology, ul. Wólczańska 213, 93-005 Łódź, Poland; anna.kowalska.1@p.lodz.pl (A.Ś.); marcin.bizukojc@p.lodz.pl (M.B.)

**Keywords:** *Penicillium rubens*, *Streptomyces noursei*, bioreactor, secondary metabolites, co-culture

## Abstract

The stirred tank bioreactor co-cultures of the filamentous fungus *Penicillium rubens* and actinomycete *Streptomyces noursei* were studied with regard to secondary metabolite (SM) production, sugar consumption, and dissolved oxygen levels. In addition to the quantitative analysis of penicillin G and nystatin A1, the broad repertoire of 22 putatively identified products was semi-quantitatively evaluated with the use of UPLC-MS. Three co-cultivation variants differing with respect to the co-culture initiation method (i.e., the simultaneous inoculation of *P. rubens* and *S. noursei* and the 24 or 48 h inoculation delay of *S. noursei* relative to *P. rubens*) were investigated. All the co-cultures were carried out in parallel with the corresponding monoculture controls. Even though *S. noursei* showed the tendency to outperform *P. rubens* and inhibit the production of fungal secondary metabolites, the approach of simultaneous inoculation was effective in terms of enhancing the production of some *S. noursei* SMs, namely desferrioxamine E, deshydroxynocardamine, and argvalin. *S. noursei* displayed the capability of adaptation and SM production even after being inoculated into the 24 or 48 h culture of *P. rubens*. Interestingly, *S. noursei* turned out to be more efficient in terms of secondary metabolite production when its inoculation time relative to *P. rubens* was delayed by 48 h rather than by 24 h. The study demonstrated that the prolongation of inoculation delays can be beneficial for production-related performance in some co-culture systems.

## 1. Introduction

Secondary metabolites (SMs), also referred to as natural products or specialized metabolites, constitute an important group of biotechnological products. The relevance of SMs is primarily of a pharmaceutical nature, as it is not uncommon for SMs to display bioactivities relevant in the context of drug discovery initiatives [1,2]. Examples of pharmaceutically relevant SMs include penicillin, an antibiotic discovered by Alexander Fleming, and lovastatin, a cholesterol-lowering drug. In relation to public health security, it is vital to address emerging threats such as the antibiotic resistance of pathogens and the risk of pandemic outbreaks. Unsurprisingly, the need for novel medicines remains unabated [3,4]. While it is now possible to generate chemical libraries of potential drug leads by employing combinatorial approaches, the screening of remarkably rich and largely unexplored biosynthetic repertoires of microorganisms still provides an attractive alternative to synthetic methods. The structural complexity of natural products and the difficulty of designing economically viable synthetic routes to synthesize these molecules are crucial points to consider in drug discovery and development [5,6,7]. On the other hand, uncovering the full biosynthetic potential of microorganisms is not a straightforward task. The production of SMs is strictly dependent on environmental conditions and, in contrast to the group of primary metabolites, is not indispensable in terms of sustaining life. To reveal the full SM catalog of a given microbial strain, one may resort to genetic manipulations or follow the bioprocess-based strategy of using unconventional methods of cultivation [8,9]. Microbial co-cultures serve this purpose ideally, as they set up the conditions for interspecies interactions that may ultimately lead to the awakening of metabolic pathways that are inactive in monocultures [10,11,12,13,14,15]. For some of the biosynthetic pathways, the stimulatory or inhibitory effects may be observable under the co-cultivation condition, as reflected, respectively, by the increased or decreased levels of SMs in the medium. Notably, the transition from mono- to co-cultivation does not require additional equipment costs unless a dedicated (e.g., membrane-based) system is required to separate the proliferating strains. Such efforts are undertaken to determine if the physical contact of the strains is necessary for SM induction. In other cases, however, the co-cultures can be successfully propagated on agar plates or in conventional laboratory flasks and bioreactors [16,17,18]. It is important to emphasize the fact that the challenges of microbial co-cultivation are mainly associated with experimental design since the requirements and characteristics of all employed strains must be taken into consideration. One of the unwanted scenarios occurs when a faster-growing microbe exerts its dominance over a slower-growing partner and practically eliminates it from the co-culture [19]. As a result, the SMs of only one strain are detected in the medium, and, depending on the research goals, the purpose of performing such co-cultivation experiments may be seen as questionable. To circumvent this problem, one may adjust the inoculum ratio or delay the inoculation of the faster-growing microorganism to allow the “slow grower” to develop its biomass prior to the moment of confrontation. The problem is that the bioreactor-based production of SMs in microbial co-cultures is greatly underexplored; the stirred tank bioreactor-based studies on SM production are still scarce [20,21,22,23], while the majority of co-cultures are investigated with the use of shake flasks. It seems surprising since the industrial production of biotechnologically relevant microbial SMs is mostly performed in stirred tank bioreactor systems. This topic is directly addressed in the present work, which is the first investigation of the stirred tank bioreactor co-cultures of *Penicillium rubens* and *Streptomyces noursei*, two potent and biotechnologically relevant producers of SMs. The filamentous fungus *P. rubens* is mostly known for its ability to biosynthesize penicillin [24], the first antibiotic produced on an industrial scale, while *S. noursei* is an actinomycete equipped with the biosynthetic pathways leading to nystatin A1 [25], a commonly prescribed antifungal drug. Furthermore, the repertoires of SMs exhibited by these two species encompass a broad array of molecules, including cycloheximide [26] and chrysogine [27], the molecules generated by *S. noursei* and *P. rubens*, respectively. As with any submerged co-cultivation process aimed at SM biosynthesis, it is of fundamental importance to study the impact of the co-culture initiation method on the outcomes of the bioprocess. Specifically, it is crucial to investigate the SM-related effects of the simultaneous inoculation of two strains as well as the delayed inoculation of the faster-growing strain. Moreover, the comparative analysis involving the co-cultures and the corresponding monocultures provides valuable information regarding the stimulatory or inhibitory effects associated with co-cultivation. Finally, it should be emphasized that each pair of co-cultivated microbial strains constitutes a unique biological system whose behavior cannot be predicted on entirely theoretical grounds. Hence, the performance of the co-culture in relation to the chosen inoculation routine must be investigated experimentally.

The aim of the study was to characterize the production of SMs in the stirred tank bioreactor co-cultures of *P. rubens* and *S. noursei*. The co-cultures initiated by performing the simultaneous inoculation of *P. rubens* and *S. noursei* were compared with the ones started by employing the delayed inoculation of *S. noursei* relative to *P. rubens*. All the investigated co-cultures were compared with their monoculture counterparts.

## 2. Materials and Methods

### 2.1. Strains

The strains *Penicillium rubens* ATCC 28089 and *Streptomyces noursei* ATCC 11455, purchased from the American Type Culture Collection (ATCC, Manassas, VA, USA), were used in the study. The strains were maintained on agar slants, as recommended by the ATCC.

### 2.2. Cultivation Medium

The following liquid medium composition was used: glucose, 10 g L^−1^; lactose, 40 g L^−1^; yeast extract, 10 g L^−1^; KH_2_PO_4_, 1.51 g L^−1^; MgSO_4_·7H_2_O, 0.5 g L^−1^; NaCl, 0.4 g L^−1^; ZnSO_4_·7H_2_O, 1 g L^−1^; Fe(NO_3_)_3_·9H_2_O, 2 g L^−1^; biotin, 0.04 mg L^−1^; and phenylacetic acid, 0.25 g L^−1^. The following solution, added at 1 mL L^−1^, was employed as a source of trace metals: MnSO_4_, 50 mg L^−1^; Na_2_B_4_O_7_·10H_2_O, 100 mg L^−1^; CuSO_4_·5H_2_O, 250 mg L^−1^; and Na_2_MoO_4_·H_2_O, 50 mg L^−1^. The pH of the medium was adjusted to 6.5 with a 0.4 M solution of sodium and potassium carbonates prior to autoclaving at 121 °C.

### 2.3. Cultivation Conditions

The BIOSTAT^®^ B stirred tank bioreactors (Sartorius, Goettingen, Germany) with a working volume of 5.5 L were employed in the study. The following experimental runs were performed: PRSN1, PRSN2, and PRSN3. In each experimental run, three bioreactors were used in parallel because the co-culture was always accompanied by the monocultures of *P. rubens* and *S. noursei*. The dissolved oxygen (DO) concentration was not controlled. The following aeration profile was used: the airflow rate was set at 1.5 L min^−1^ in the first 24 h of the run and was increased to 5 L min^−1^ until the end of the process, while the stirring speed was constant at 300 min^−1^. All experiments were performed in triplicate, and the standard deviation values were calculated in OriginPro 2017 software (OriginLab, Version b9.4.1.354 SR1, Northampton, MA, USA).

### 2.4. Co-Culture Initiation Methods

The spores of *P. rubens* were obtained through cultivation on a solid medium prepared by adding glucose (20 g L^−1^) and agar (20 g L^−1^) to potato broth (300 g of potatoes boiled in 500 mL of water). The ISP Medium 2 (BD, Franklin Lakes, NJ, USA), used according to the manufacturer’s instructions, was employed for the preparation of *S. noursei* spores. The spore suspension, obtained by removing the spores from agar slants with the use of a sterile pipette, was applied for the inoculation of sterile production medium in the bioreactor (150 mL for the monoculture, 150 + 150 = 300 mL for the co-culture). Following the completion of the inoculation procedure, the working volume in all bioreactors (i.e., mono- and co-cultures) was always equal to 5.5 L. The inoculation procedure was adjusted to reach the final spore concentration in the bioreactor equal to (1.0 ± 0.1) × 10^9^ spores per liter. The following inoculation scheme was applied in the PRSN1, PRSN2, and PRSN3 co-cultures:

PRSN1: simultaneous inoculation of *S. noursei* and *P. rubens*;
PRSN2: *S. noursei* inoculation delayed by 24 h relative to *P. rubens*;
PRSN3: *S. noursei* inoculation delayed by 48 h relative to *P. rubens*.

### 2.5. Analytical Procedures

Liquid samples were collected from each bioreactor at 24 h intervals. The biomass was removed through filtration with the use of Munktell filter discs (grade 389.84 g m^−2^, diameter 150 mm). The liquid samples were frozen at −20 °C.

For the analysis of SMs in the liquid samples, ultra-high-performance liquid chromatography (AQUITY-UPLC^®^) coupled with high-resolution mass spectrometry (ACQUITY–SYNAPT G2, Waters, Milford, MA, USA) was employed, as described in detail in [23]. The column BEH Shield RP18 (reverse-phase), 2.1 mm × 100 mm × 1.7 μm, was used for SM analysis. For the analysis of glucose and lactose, the BEH Amide column (normal phase), 2.1 mm × 150 mm × 1.7 μm, coupled with an evaporative light scattering detector (Waters, Milford, MA, USA) was employed, as described in detail in [23].

The identification of metabolites was based on the comparison of the experimental *m*/*z* values (at ESI^−^ mode) with the database records of The Natural Product Atlas [28]. The identities and concentrations of penicillin G and nystatin A1 were determined using the analytical standards purchased from Sigma-Aldrich (Burlington, MA, USA). In the cases of the remaining SMs, for which the standards were not available, the identity was putatively assigned based upon the *m*/*z* similarity to the previously described *Streptomyces* products (with the absolute error Δ*m*/*z* always below the value of 0.01) included in The Natural Product Atlas [28], and the peak area representing the [M−H]^−^ ion was considered for semi-quantitative comparisons.

The volumetric uptake rates of glucose (r_GLU_) and lactose (r_LAC_) were obtained by approximating the concentration values with cubic b-spline function and differentiating the curves in time. PTC Mathcad 15 (PTC, Version 15.0, Boston, MA, USA) software was applied for this purpose.

The light microscope OLYMPUS BX53 (Olympus Corporation, Tokyo, Japan) with the software OLYMPUS cellSens Dimension Desktop 1.16 (Olympus Corporation, Version 1.16, Tokyo, Japan) was employed for the microscopic observations.

## 3. Results

### 3.1. Production of Secondary Metabolites

**Figure 1:**
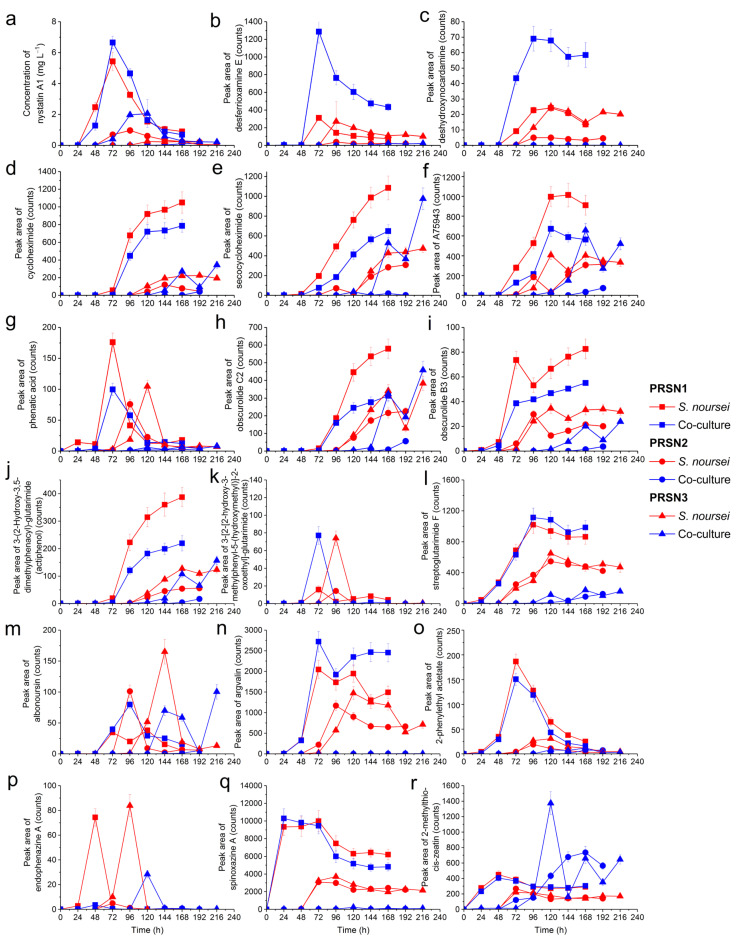
Time courses of secondary metabolite levels in the co-cultures of *P. rubens* and *S. noursei* and the corresponding monocultures of *S. noursei*. The production profiles of 18 metabolites originating from *S. noursei* are depicted, including nystatin A1 (**a**) and the putatively identified desferrioxamine E (**b**), deshydroxynocardamine (**c**), cycloheximide (**d**), secocycloheximide (**e**), A75943 (**f**), phenatic acid A (**g**), obscurolide C2 (**h**), obscurolide B3 (**i**), 3-(2-hydroxy-3,5-dimethylphenacyl)glutarimide (actiphenol) (**j**), 3-[2-[2-hydroxy-3-methylphenyl-5-(hydroxymethyl)]-2-oxoethyl]glutarimide (**k**), streptoglutarimide F (**l**), albonoursin (**m**), argvalin (**n**), 2-phenylethyl acetate (**o**), endophenazine A (**p**), spinoxazine A (**q**), and 2-methylthio-cis-zeatin (**r**). Error bars represent ±standard deviation values calculated for experimental triplicates.

**Figure 2:**
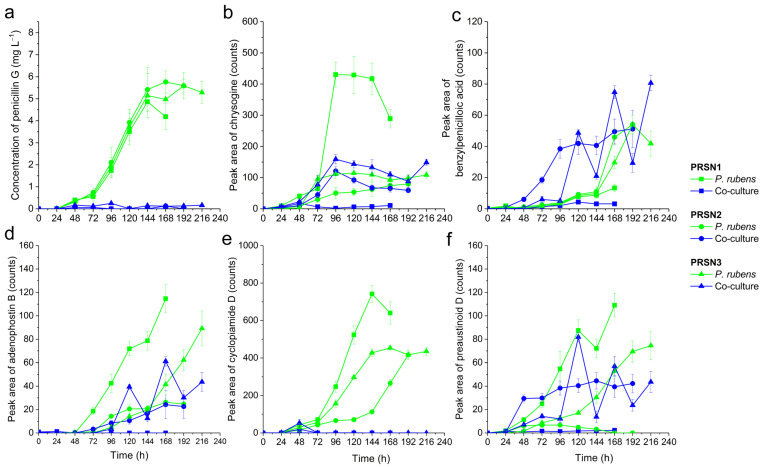
Time courses of secondary metabolite levels in the co-cultures of *P. rubens* and *S. noursei* and the corresponding monocultures of *P. rubens*. The production profiles of 6 metabolites originating from *P. rubens* are depicted, including penicillin G (**a**) and the putatively identified chrysogine (**b**), benzylpenicilloic acid (**c**), adenophostin B (**d**), cyclopiamide D (**e**), and preaustinoid D (**f**). Error bars represent ±standard deviation values calculated for experimental triplicates.

The analysis of cultivation broth samples resulted in the detection of 24 SMs, including 18 and 6 molecules originating from *S. noursei* and *P. rubens*, respectively (Table 1). The identities and concentration values of the two industrially relevant products biosynthesized using these microorganisms, namely nystatin A1 and penicillin G, were determined by employing the commercially available authentic standards. Due to the lack of reference standards, the remaining SMs were putatively annotated by comparing their experimental *m/z* values with the database records for the previously discovered SMs of the same or related species, as detailed in the Materials and Methods section. In addition, the total ion chromatograms (TICs) were recorded for every collected sample (Figures S1–S27) to have a “bird’s eye view” of the composition changes of the broth over the course of the cultivation. Most importantly, the levels of SMs originating from *S. noursei* (Figure 1) and *P. rubens* (Figure 2) were determined and compared among the tested experimental variants.

As far as the SMs of *S. noursei* were concerned, their production profiles in the co-cultures differed markedly depending on the experimental run (Figure 1). In the PRSN1 co-culture, which was simultaneously inoculated with the spore suspensions of *S. noursei* and *P. rubens*, the presence of SMs (e.g., nystatin A1, see Figure 1a) was detected as early as 48 h of the co-cultivation process. In the PRSN2 and PRSN3 co-cultures, for which the *S. noursei* inoculation was delayed, respectively, by 24 or 48 h relative to *P. rubens*, the presence of most *S. noursei* SMs was typically detected much later, i.e., 120 or 72 h after *S. noursei* inoculation in the PRSN2 and PRSN3 runs, respectively. It was also noticed that the vast majority of *S. noursei* products became detectable in the PRSN3 co-culture at earlier times and at higher levels than in the PRSN2 co-culture (e.g., see Figure 1a,d,f,i).

The impact of co-cultivation varied among the analyzed SMs. Initially, it was observed that the highest mean levels of 8 out of 18 SMs of *S. noursei* were recorded not for the monocultures but for their co-culture counterparts, including nystatin A1, desferrioxamine E, deshydroxynocardamine, 3-[2-[2-hydroxy-3-methylphenyl-5-(hydroxymethyl)]-2-oxoethyl]glutarimide, streptoglutarimide F, argvalin, spinoxazine A, and 2-methylthio-cis-zeatin. However, after careful consideration of all the production profiles and experimental errors, it became evident that the marked stimulatory effects exerted via co-cultivation were confirmed only for a small number of SMs. For instance, one could clearly observe the enhancement of desferrioxamine E (Figure 1b) and deshydroxynocardamine (Figure 1c) production in the PRSN1 co-culture relative to the PRSN1 monocultures of *P. rubens* and *S. noursei*. Notably, the improved production of these two siderophore SMs was not universally displayed in all co-culture bioprocesses, i.e., the PRSN2 and PRSN3 co-cultures turned out to be strongly inhibitory in terms of desferrioxamine E and deshydroxynocardamine biosynthesis (Figure 1b,c). A similar observation was made with regard to the production of argvalin, which was boosted in the PRSN1 co-culture but not in the PRSN2 and PRSN3 counterparts (Figure 1n). By contrast, the formation of 2-methylthio-cis-zeatin was stimulated in the PRSN2 and PRSN3 co-cultures, albeit not throughout the whole duration of the process (Figure 1r). The production of secocycloheximide (Figure 1e), A75943 (Figure 1f), and obscurolide C2 (Figure 1h) was temporarily enhanced in the PRSN3 co-culture relative to the PRSN3 monoculture of *S. noursei*; however, this phenomenon did not take place in the PRSN1 and PRSN2 experiments. In the case of obscurolide B3 (Figure 1i), the production levels observed in the PRSN1, PRSN2, and PRSN3 co-cultures were lower than the ones noted for the corresponding *S. noursei* monocultures. At this point, one should also mention the co-culture-related inhibitory effects recorded for some of the identified SMs. Such behavior was noticed for several products of *S. noursei*, including cycloheximide (Figure 1d), phenatic acid (Figure 1g), and actiphenol (Figure 1j).

As far as the molecules originating from *P. rubens* were concerned, for all the products except benzylpenicilloic acid, the highest mean levels were recorded in the monocultures (Figure 2). Hence, considering the production profiles of all the identified *P. rubens* metabolites, the predominant behavior was the inhibition of SM biosynthesis under the conditions of co-cultivation. This was evident, especially in the case of penicillin G (Figure 2a). The production of this antibiotic was practically turned off in the co-cultures (Figure 2a). A similar blocking effect was noted for cyclopiamide D (Figure 2e). Even though for chrysogine (Figure 2b), adenophostin B (Figure 2d), and preaustinoid D (Figure 2f), the inhibitory effects of co-cultivation were not as strong as for penicillin G and cyclopiamide D, it was clear that the monocultures led to visibly higher production levels of these SMs. Another observation made with regard to the SMs of *P. rubens* was that their highest mean levels reached in PRSN2 co-culture were generally lower than the highest mean values recorded for the PRSN3 co-culture (Figure 2), similarly as was observed for the products of *S. noursei* (Figure 1).

### 3.2. Changes in Dissolved Oxygen Levels

**Figure 3:**
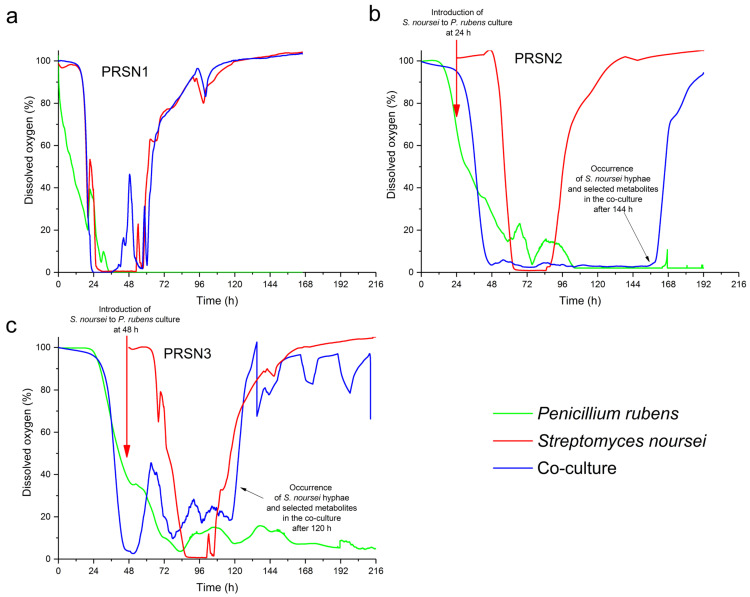
Dissolved oxygen (DO) levels in the PRSN1 (**a**), PRSN2 (**b**), and PRSN3 (**c**) co-cultures of *P. rubens* and *S. noursei* and the corresponding monocultures.

The dissolved oxygen (DO) profiles recorded for the co-cultures were compared with the ones displayed by the monocultures of *P. rubens* and *S. noursei* in the PRSN1 (Figure 3a), PRSN2 (Figure 3b), and PRSN3 (Figure 3c) runs. The typical behavior observed in the *S. noursei* monocultures (red lines in Figure 3) was that the DO level went down to zero within the initial 24–36 h after inoculation, then remained at the zero level for approximately 24 h and, finally, increased asymptotically back towards the 100% level line. Compared with *S. noursei*, the DO curves in the *P. rubens* monocultures (black lines in Figure 3) decreased less rapidly towards the zero line following the inoculation and did not show an increase even after 168 h of the cultivation process. For the co-cultures (blue lines in Figure 3), the shapes of DO profiles varied among the experimental runs. The DO curve recorded for the PRSN1 co-culture resembled the one exhibited by the corresponding PRSN1 monoculture of *S. noursei* for almost the entire period of the cultivation (Figure 3a). In the case of the PRSN2 run (Figure 3b), the co-culture curve partially resembled *S. noursei* and *P. rubens* monoculture curves, albeit at different process times. In the initial period (i.e., within 48 h after *P. rubens* inoculation or, correspondingly, 24 h after *S. noursei* inoculation), the shape of the DO profile of the PRSN2 co-culture was similar to the curve displayed by the corresponding *S. noursei* monoculture. Then, within the interval between 48 and 156 h, after *P. rubens* inoculation, the DO level of the co-culture was close to zero. Such a prolonged time of strong oxygen consumption was typically noted for the individual growth of *P. rubens*, as reflected by the DO curve in the corresponding PRSN2 fungal monoculture (Figure 3b). In PRSN2, the similarity between the co-culture and *P. rubens* monoculture lasted for 156 h after the time of *P. rubens* inoculation. Afterwards, the DO profiles of *P. rubens* monoculture and the “*P. rubens* vs. *S. noursei*” co-culture ceased to overlap, and the co-culture DO level started to increase towards the 100% line in a similar abrupt fashion as seen in the *S. noursei* monoculture (Figure 3b).

In the case of the DO curve in the PRSN3 co-culture (Figure 3c), the increase in DO concentration started earlier than in the PRSN2, i.e., already 120 h after *P. rubens* inoculation or, if the actinomycete inoculation is chosen as a reference time point, 72 h after *S. noursei* introduction into the bioreactor. It is important to note that the time interval ranging from *S. noursei* inoculation to the moment when the DO profiles reached an inflection point (and hereby started to rise towards 100%) varied markedly among the three investigated co-cultures. To be more specific, this period lasted for 60, 132, and 72 h in PRSN1, PRSN2, and PRSN3 co-cultures, respectively.

### 3.3. Consumption of Lactose and Glucose

**Figure 4:**
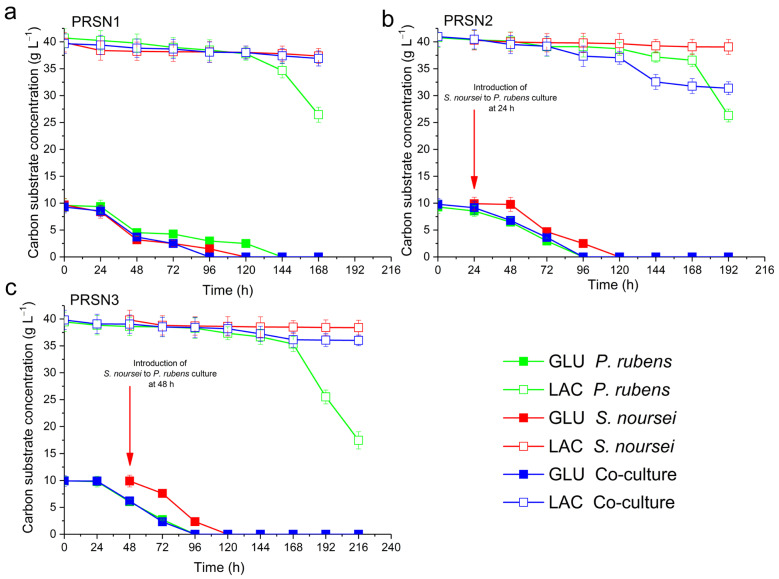
Glucose (GLU) and lactose (LAC) concentration values in the PRSN1 (**a**), PRSN2 (**b**), and PRSN3 (**c**) co-cultures of *P. rubens* and *S. noursei* and the corresponding monocultures. Error bars represent ±standard deviation values calculated for experimental triplicates.

The time courses of glucose and lactose concentration (Figure 4) revealed that there was practically no lactose consumption in *S. noursei* monocultures. Since *S. noursei* proved to be incapable of lactose utilization, it was clear that any decrease in lactose concentration or changes in lactose uptake rates in the co-cultures could be attributed solely to *P. rubens*.

In the PRSN1 coculture (Figure 4a), which was inoculated simultaneously with *P. rubens* and *S. noursei* spores, no lactose uptake was observed. It was also noticed that the co-culture curve closely resembled the one recorded for the monoculture of *S. noursei*. Hence, according to the data, *P. rubens* was either absent or did not consume lactose in the PRSN1 co-culture. By contrast, lactose concentration in *P. rubens* monoculture did not show any changes until 120 h, but then it went down to 25 g L^−1^ within the time interval between 120 and 168 h of the process. In the case of glucose consumption (Figure 4a), the concentration profiles were almost identical for *S. noursei* monoculture and co-culture, for which the exhaustion of glucose was evident at 120 h. In *P. rubens* monoculture, glucose was no longer detectable at 144 h.

**Figure 5:**
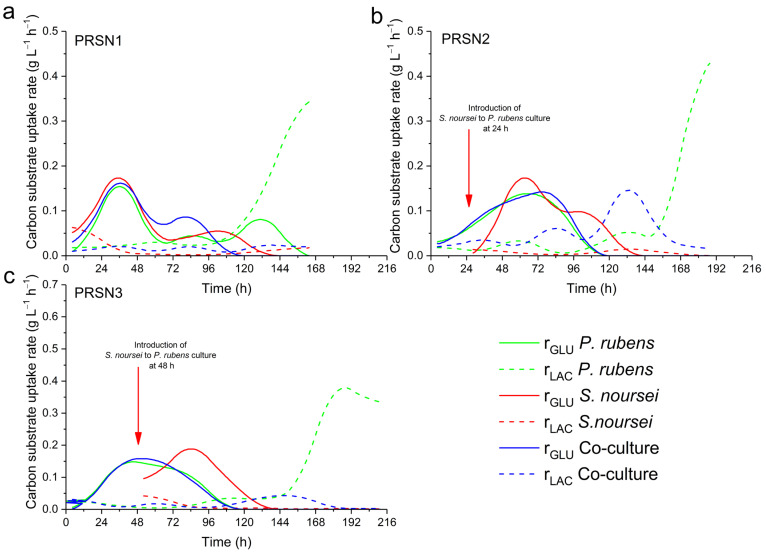
Volumetric uptake rate of glucose (r_GLU_) and lactose (r_LAC_) levels in the PRSN1 (**a**), PRSN2 (**b**), and PRSN3 (**c**) co-cultures of *P. rubens* and *S. noursei* and the corresponding monocultures.

An interesting observation was made with regard to the PRSN2 co-culture (Figure 4b), in which more lactose was consumed in the co-culture than in the monoculture of *P. rubens*, with the exception of the last day of the process, when an abrupt decrease in lactose level occurred in the monoculture variant. In the PRSN3 co-culture (Figure 4c), on the other hand, the consumption of lactose was recorded, but it did not proceed according to the same scenario as in PRSN2. Specifically, the lactose concentration curves of the PRSN3 co-culture and *P. rubens* monoculture practically overlapped until 168 h after *P. rubens* inoculation, and then the utilization of lactose ceased in the co-culture, while the lactose level in *P. rubens* monoculture decreased steeply (Figure 4c). Overall, the use of lactose in the PRSN3 (Figure 4c) co-culture was far less intensive than that in the PRSN2 counterpart (Figure 4b), as indicated by the relatively high concentration of lactose still available in the medium after the PRSN3 co-cultivation process was terminated. As far as glucose utilization was concerned in the PRSN2 and PRSN3 runs, there was a visible overlap between the concentration curves recorded for the co-culture and *P. rubens* monoculture (Figure 4b,c). It may be argued, however, that the consumption of glucose in the *S. noursei* monoculture also showed some similarities with the *P. rubens* and co-culture curves. By considering the sugar concentration-related dataset for the PRSN1, PRSN2, and PRSN3 runs (Figure 4), it is well-grounded to state that the investigated mono- and co-cultures were more distinguishable in terms of lactose consumption profiles than with regard to glucose utilization curves. Similar observations were made when the volumetric substrate uptake rates were calculated (Figure 5).

The monocultures of *P. rubens* clearly stood out in terms of lactose utilization rates when compared to other variants. More specifically, the recorded r_LAC_ values for the *P. rubens* monocultures were not only increasing towards the end of the cultivation period, but they also reached considerably higher levels relative to the co-cultures and *S. noursei* monocultures (Figure 5). Another important observation was that within the period from 70 to 150 h after *P. rubens* inoculation, the r_LAC_ values in the PRSN2 co-culture were clearly above the curve representing the *P. rubens* monoculture (Figure 5b). Importantly, such behavior was not recorded in the PRSN1 and PRSN3 co-cultures.

## 4. Discussion

The study represents a multi-angle investigation of stirred tank bioreactor co-cultures involving *S. noursei* and *P. rubens*. What emerges from the experimental datasets is a description of three distinct co-cultivation scenarios. It is convenient to start the discussion by considering the similarities between the DO profiles recorded for the mono- and co-cultures (Figure 3). A similar pattern was observed in all three experimental runs for the monocultures of *S. noursei*, i.e., a quick decrease to 0% after the inoculation, then the 24 h period at 0%, and, finally, the inflection back towards the 100% level. The question was whether the DO profiles recorded for the PRSN1, PRSN2, and PRSN3 co-cultures followed the same behavior as noted for the *S. noursei* monocultures. In the case of the PRSN1 co-cultivation variant, the DO curve practically overlapped with the *S. noursei* monoculture line (Figure 3a). In PRSN2 co-culture, in which *S. noursei* was inoculated 24 h after *P. rubens*, the DO curve remained at the 0% level much longer than in PRSN1, i.e., for almost 110 h. The difference between PRSN1 and PRSN2 in terms of oxygen consumption was thus striking. Finally, the DO behavior in the PRSN3 co-culture was unique in two aspects. Firstly, the period from the moment of reaching the 0% level to inflecting back towards the saturation line lasted for about 72 h, i.e., it was longer than in the *S. noursei* monoculture but, at the same time, not as long as in the PRSN2 co-culture. Secondly, the DO profile in the co-culture displayed a somewhat fluctuating trend following its descent to 0%, i.e., it remained at 0% only temporarily, in contrast to what was seen in the PRSN1 and PRSN2 co-cultivations. The DO curves indicated that the PRSN1 co-culture resembled the *S. noursei* monoculture and that the simultaneous inoculation of the two microbes did not provide favorable conditions for the development of *P. rubens* biomass, which was in turn associated with the company of a fast-growing *S. noursei*. In other words, the fungus was dominated by the actinomycete, and the proliferation of *P. rubens* mycelium was greatly inhibited under such circumstances. This observation was confirmed during the analysis of SMs originating from *P. rubens* (Figure 2). They were either absent or present in trace amounts in the PRSN1 co-culture. Another piece of evidence was provided when sugar concentrations were determined (Figure 4). The lack of visible lactose consumption in the PRSN1 co-culture indicated that *P. rubens* growth was blocked. The TICs (Figures S1–S8) collected over the course of the PRSN1 run agreed well with the hypothesis of the domination of *S. noursei* over *P. rubens* in the simultaneously inoculated co-culture because the data representing the co-culture and the *S. noursei* monoculture were highly similar. In the PRSN2 and PRSN3 co-cultivation variants, in which the inoculation of *S. noursei* was delayed, the biomass of *P. rubens* could freely develop under monoculture conditions for 24 and 48 h, respectively. The main question at this point was if *S. noursei* could propagate in the presence of the already-developed biomass of *P. rubens*. According to the results of the study, *P. rubens* did not prevent *S. noursei* from proliferating in the co-culture; however, the presence of the actinomycete was not manifested until the late phases of co-cultivation, as demonstrated with the microscopic analysis and the UPLC-MS assays. Firstly, the microscopic images (Figures S28–S30) revealed the existence of *S. noursei* biomass in the cultivation broth of PRSN2 and PRSN3 co-cultivation. Secondly, the SMs of *S. noursei* became detectable in the late phases of the PRSN2 and PRSN3 co-cultures (Figure 1 and Figures S10–S27), which illustrated the “awakening” of the biosynthetic activity of the actinomycete. Finally, the consumption of lactose stopped in the final stages of these co-cultures (Figure 4), which probably corresponded to the inhibition of *P. rubens* by *S. noursei*. It should also be mentioned that the DO profiles of PRSN2 and PRSN3 co-cultures showed inflection points towards the 100% level, which was a characteristic feature of *S. noursei* monocultures. However, it took considerably more time for the co-cultures to reach the moment of DO inflection compared with the *S. noursei* monoculture variants (Figure 3). All in all, the results indicated that despite the domination of *S. noursei* over *P. rubens*, the former eventually came into play in the PRSN2 and PRSN3 co-cultures despite the growth-related advantage granted to the fungus by delaying the inoculation of the actinomycete.

Even though both PRSN2 and PRSN3 co-cultivation variants were based on the concept of delayed inoculation of *S. noursei* to enable the growth of *P. rubens*, they differed with respect to the time of delay. According to the results, the co-culture initiation approach had profound effects on the outcomes of the studied bioprocess, as reflected by the levels of SMs reached in the PRSN2 and PRSN3 co-cultures (Figure 1 and Figure 2). Perhaps the best illustration of the differences between these two co-cultures was provided through DO measurements (Figure 3). As mentioned before, the DO profile recorded for the PRSN2 co-culture was characterized by a relatively long period of 0% oxygen level prior to displaying the behavior reminiscent of the *S. noursei* monocultures, namely the inflection of the DO curve towards the 100% line. Such a scenario could be attributed to the fact that *S. noursei* in the PRSN2 co-culture required more time for biomass development and for exerting its domination over *P. rubens* in comparison with the PRSN3 counterpart, in which the time from *S. noursei* inoculation to the characteristic inflection point was markedly shorter than in PRSN2. It all demonstrated that the events taking place during “*S. noursei* vs. *P. rubens*” co-cultivation were, at least to a certain degree, determined using the co-culture initiation approach. This leads to the question of the possible reasons for the differences between PRSN2 and PRSN3. They might have been associated with the development and changing characteristics of fungal pellets under submerged conditions. The pellets are not static units; their structures undergo changes as the cultivation progresses. For instance, the biomass growth takes place not only on the exterior of the pellet but also within its core region. While this topic has not yet been investigated, it is justified to assume that the ability of *P. rubens* biomass to engulf (or “trap”) the spores of *S. noursei* inside fungal mycelium changes over cultivation time, in concert with the structural transformations of *P. rubens* pellets. In other words, the differences related to *P. rubens* biomass age in PRSN2 and PRSN3 co-cultures were accompanied by differences with regard to the structural features of fungal pellets and, consequently, their capabilities to inhibit *S. noursei* development. Quite surprisingly, the recorded results contradicted the intuitive presumption that the longer delay of *S. noursei* inoculation (i.e., 48 h in PRSN3) would result in a longer period of *S. noursei* adaptation and, possibly, an even more delayed onset of the domination exerted by *S. noursei* over *P. rubens*. In fact, the levels of *S. noursei* SMs products were usually higher in PRSN3 than in PRSN2, their presence in the PRSN3 broth was typically confirmed after shorter post-inoculation periods than in PRSN2 (Figure 1), and the inflection of the DO profile came much sooner in PRSN3 compared with PRSN2 (Figure 3). In short, *S. noursei* seemed to perform more efficiently in PRSN3, in which the actinomycete inoculation delay relative to *P. rubens* (i.e., 48 h) was longer than in PRSN2 (i.e., 24 h). Another observation that seemed counterintuitive was the fact that the highest levels of several SMs of *P. rubens* recorded in the co-cultures were reached not in the PRSN2 co-culture but in the PRSN3 counterpart (Figure 2b–d,f). It was surprising, as the lactose uptake rates were visibly higher in the PRSN2 co-culture than in the corresponding PRSN3 variant (Figure 4), and, as already mentioned, lactose consumption was attributed solely to *P. rubens*. Supposedly, the mere fact of vigorous lactose utilization was not associated with the marked enhancement of fungal SM production. It seemed as if the relatively fast development of *S. noursei* in the PRSN3 co-culture somehow stimulated several biosynthetic pathways in *P. rubens* cells compared with the PRSN2 co-culture, i.e., the biochemical routes leading to chrysogine (Figure 2b), benzylpenicilloic acid (Figure 2c), adenophostin B (Figure 2d), and preaustinoid D (Figure 2f).

The fact that *S. noursei* was able to grow and produce SMs after being introduced into the already developed *P. rubens* culture agreed with the previous work on “*Streptomyces rimosus* vs. *P. rubens*” co-cultivation [23], in which the actinomycete eventually showed its dominance over the fungus despite the inoculation-related disadvantage (i.e., a 24 or 48 h delay). Once more, the utilization of a substrate selectively consumed solely by one of the co-cultivated strains, namely lactose, proved to be an effective method of co-culture investigation. The present study also confirmed the importance of the co-culture initiation strategy for the outcomes of the microbial co-cultivation process. What made the “*S. noursei* vs. *P. rubens*” system unique was the fact that prolonging the inoculation delay of the actinomycete from 24 (as in PRSN2) to 48 h (as in PRSN3) was often beneficial for the co-culture in the context of SM production, especially with regard to the metabolites originating from *S. noursei*. The previously studied “*S. rimosus* vs. *P. rubens*” co-cultivation did not reveal such tendencies [23]. All in all, it is well-grounded to state that the “*S. noursei* vs. *P. rubens*” and “*S. rimosus* vs. *P. rubens*” co-cultures did not follow the same path, even though they shared some characteristics related to the survival of *Streptomyces* sp. in the filamentous biomass of *P. rubens*. It should be emphasized that the axenic culture was demonstrated to be a preferred system of production for the majority of *S. noursei* and *P. rubens* SMs identified in the present study. Still, the “*S. noursei* vs. *P. rubens*” co-culture was shown to be worth considering as an alternative to the axenic culture in the context of the production of several SMs, most notably desferrioxamine E and deshydroxynocardamine.

Despite the fact that the PRSN1 co-culture and the *S. noursei* monoculture were highly similar in terms of DO profiles and sugar consumption, they were far from being equivalent in terms of SM production (Figure 1). For instance, the levels of desferioxamine E, deshydroxynocardamine, and argvalin were elevated in the PRSN1 co-culture relative to the *S. noursei* variant. On the other hand, in the case of actiphenol and obscurolide C2, the PRSN1 co-culture resulted in visibly weaker production than the one recorded for *S. noursei* monocultivation. So, the presence of *P. rubens* could not be regarded as negligible, even though the production of SMs was practically non-existent during PRSN1 co-cultivation owing to the domination of *S. noursei*. In other words, even the blocked growth of *P. rubens* did not prevent the fungus from affecting the SM production via *S. noursei*. However, the mechanism responsible for this effect remains unknown, and further investigation would be required to elucidate its nature. Finally, it should be underscored that the stimulation of SM biosynthesis was associated with the inoculation scheme; e.g., the aforementioned enhancement of desferrioxamine E, deshydroxynocardamine, and argvalin production occurred exclusively in PRSN1.

The co-cultivation of *Penicillium* with *Streptomyces* was previously investigated in the context of SM induction in laboratory flasks. In a study by Wang et al. [29], five unique SMs were obtained in the co-cultures of *Penicillium* sp. WC-29-5 and *Streptomyces fradiae* 007. More recently, Krespach et al. [30] showed that arginoketides generated by *Streptomyces* sp. activate the formation of secondary metabolites in *Penicillium* isolates. In a different study, the influence of *P. rubens* on the levels of *S. rimosus*-derived SMs was examined in shake flask co-cultures [31]. However, to the best of our knowledge, the only stirred tank bioreactor-based characterization of the “*Penicillium* vs. *Streptomyces*” co-culture system was previously reported by our group in relation to the *P. rubens*/*S. rimosus* case [23]. In that context, the present study involving *P. rubens* and *S. noursei* provided examples of the unique features, as well as the similarities, that may be encountered among the *Penicillium* vs. *Streptomyces* bioreactor co-cultivations.

## 5. Conclusions

The following main conclusions were formulated on the basis of the results of the study.

Firstly, when the co-culture is initiated through the simultaneous inoculation of *P. rubens* and *S. noursei*, the actinomycete outperforms the fungus, as illustrated by the blocked production of *P. rubens* SMs, the lack of lactose consumption, and the similarity of the co-culture DO profile with the DO curve displayed by *S. noursei* monoculture. Still, the approach of simultaneous inoculation is effective in terms of enhancing the production of some *S. noursei* SMs, namely desferrioxamine E, deshydroxynocardamine, and argvalin.

Secondly, *S. noursei* shows the capability of adaptation and SM production even after being inoculated into the 24 or 48 h culture of *P. rubens*.

Finally, *S. noursei* is more efficient in terms of SM production when its inoculation time relative to *P. rubens* is delayed by 48 h rather than by 24 h. In a broader context of submerged co-cultivation, the study demonstrated that the prolongation of the inoculation delay for a given microorganism is not necessarily detrimental to the SM production-related performance of this microorganism in the co-culture.

## Figures and Tables

**Table 1:** List of secondary metabolites identified in the bioreactor mono- and co-cultures of *P. rubens* and *S. noursei*.

Retention Time (min)	Experimental (*m*/z), ESI^−^	Suggested Formula, [M−H]^−^	Suggested Metabolite	Theoretical (*m*/z) of Suggested Metabolite, ESI^−^	Δ(*m*/z) = Experimental (*m*/z)—Theoretical (*m*/z)	Confidence Level of Metabolite Identification	Producer
6.5	333.0928	C_16_H_17_N_2_O_4_S	Penicillin G	333.0909	+0.0019	Identified	*P. rubens*
5.7	351.0989	C_16_H_19_N_2_O_5_S	Benzylpenicilloic acid	351.1015	−0.0026	Putatively annotated	*P. rubens*
4.7	189.0756	C_10_H_9_N_2_O_2_	Chrysogine	189.0665	+0.0091	Putatively annotated	*P. rubens*
8.3	491.2644	C_27_H_39_O_8_	Preaustinoid D	491.2645	−0.0001	Putatively annotated	*P. rubens*
6.5	335.1042	C_19_H_15_N_2_O_4_	Cyclopiamide D	335.1031	+0.0011	Putatively annotated	*P. rubens*
4.9	710.0464	C_18_H_27_N_5_O_19_P_3_	Adenophostin B	710.0513	−0.0049	Putatively annotated	*P. rubens*
5.9	924.4999	C_47_H_74_O_17_N	Nystatin A1	924.4957	+0.0042	Identified	*S. noursei*
4.9	601.3576	C_27_H_49_O_9_N_6_	Desferrioxamine E	601.3561	+0.0015	Putatively annotated	*S. noursei*
4.8	585.3549	C_27_H_49_O_8_N_6_	Deshydroxynocardamine	585.3612	−0.0063	Putatively annotated	*S. noursei*
5.3	280.1520	C_15_H_22_O_4_N	Cycloheximide	280.1549	−0.0029	Putatively annotated	*S. noursei*
5.9	280.1520	C_15_H_22_O_4_N	Secocycloheximide	280.1549	−0.0029	Putatively annotated	*S. noursei*
5.8	280.1520	C_15_H_22_O_4_N	A75943	280.1549	−0.0029	Putatively annotated	*S. noursei*
5.3	292.1167	C_15_H_18_ON	Phenatic acid A	291.1185	−0.0018	Putatively annotated	*S. noursei*
6.2	292.1167	C_15_H_18_ON	Obscurolide C2	291.1185	−0.0018	Putatively annotated	*S. noursei*
6.0	276.1201	C_15_H_18_O_4_N_1_	Obscurolide B3	276.1236	−0.0053	Putatively annotated	*S. noursei*
6.3	274.1042	C_15_H_16_O_4_N	3-(2-Hydroxy-3,5-dimethyl-phenacyl)glutarimide (actiphenol)	274.1079	−0.0037	Putatively annotated	*S. noursei*
6.3	290.1028	C_15_H_16_O_5_N	3-[2-[2-Hydroxy- 3-methylphenyl-5-(hydroxymethyl)]- 2-oxoethyl]glutarimide	290.1028	0.0000	Putatively annotated	*S. noursei*
4.8	310.1324	C_15_H_20_O_6_N	Streptoglutarimide F	310.1291	+0.0033	Putatively annotated	*S. noursei*
7.2	257.1300	C_15_H_17_O_2_N_2_	Albonoursin	257.1290	+0.0010	Putatively annotated	*S. noursei*
4.3	238.1678	C_11_H_20_O_1_N_5_	Argvalin	238.1668	−0.0010	Putatively annotated	*S. noursei*
7.2	163.0753	C_10_H_11_O_2_	2-Phenylethyl acetate	163.0759	−0.0006	Putatively annotated	*S. noursei*
6.5	293.1290	C_18_H_17_O_2_N_2_	Endophenazine A	293.1290	0.0000	Putatively annotated	*S. noursei*
4.7	263.1064	C_13_H_15_N_2_O_4_	Spinoxazine A	263.1032	+0.0032	Putatively annotated	*S. noursei*
4.9	264.0859	C_11_H_14_N_5_OS	2-Methylthio-cis-zeatin	264.0919	−0.0060	Putatively annotated	*S. noursei*

## Data Availability

Data will be made available on request.

## Supplementary Materials

The following supporting information can be downloaded at https://www.mdpi.com/article/10.3390/biom13121748/s1.
