# Supplementary material for: Investigating the Stirred Tank Bioreactor Co-Cultures of the Secondary Metabolite Producers Streptomyces noursei and Penicillium rubens
Source: Biomolecules. 2023 Dec 5;13(12):1748. doi: 10.3390/biom13121748 (PMC10742013; doi:10.3390/biom13121748)
Supplement: Supplementary file 1 [file biomolecules-13-01748-s001.zip › biomolecules-2748038-supplementary.pdf]

Supplementary materials

Investigating the Stirred Tank Bioreactor Co-Cultures of the Secondary Metabolite Producers *Streptomyces noursei* and *Penicillium rubens*

Tomasz Boruta *, Anna Ścigaczewska and Marcin Bizukojć

Department of Bioprocess Engineering, Faculty of Process and Environmental Engineering, Lodz University of Technology, ul. Wólczańska 213, 93-005 Łódź, Poland; anna.kowalska.1@p.lodz.pl (A.Ś.); marcin.bizukojc@p.lodz.pl (M.B.)

* Correspondence: tomasz.boruta@p.lodz.pl; Tel.: +48-42-631-39-77; Fax: +48-42-636-56-63

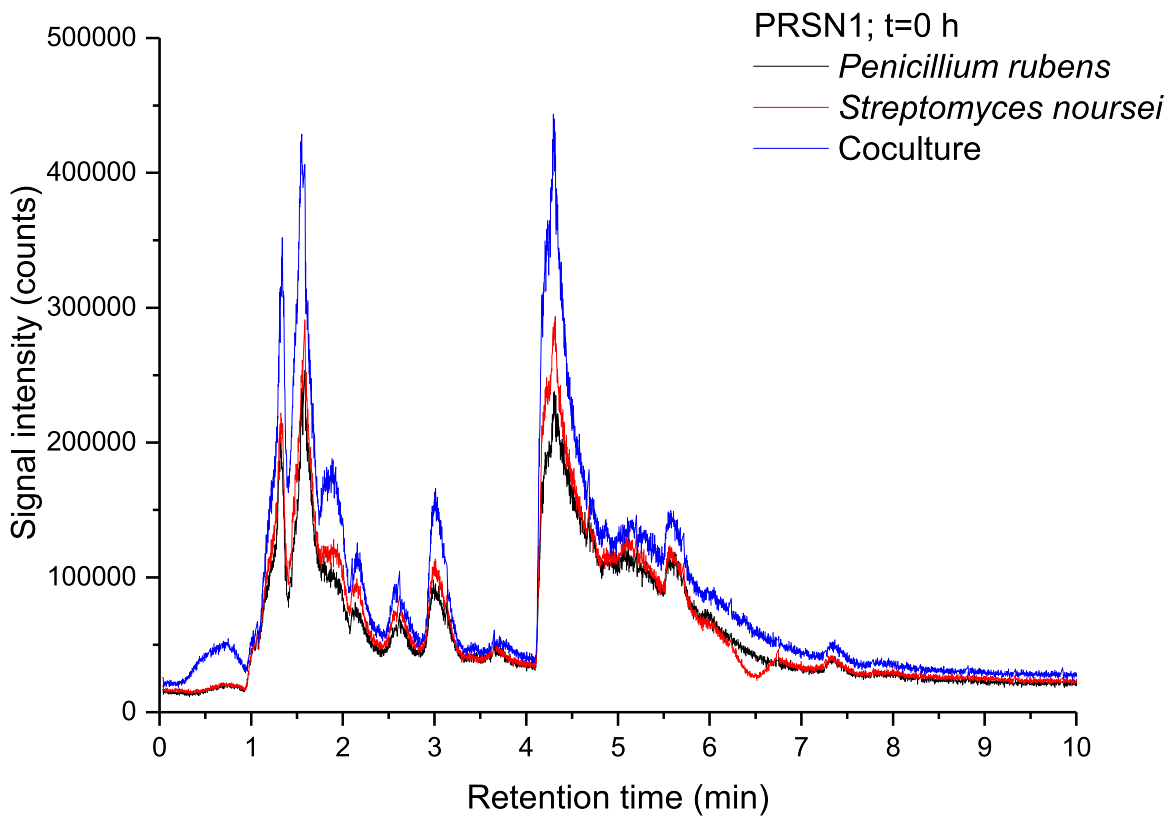

Figure S1. Alignments of total ion chromatograms (TICs) recorded in the PRSN1 experiment at t=0 h for *P. rubens* monoculture (black line), *S. noursei* monoculture (red line), and the "*P. rubens* vs. *S. noursei*" coculture (blue line)

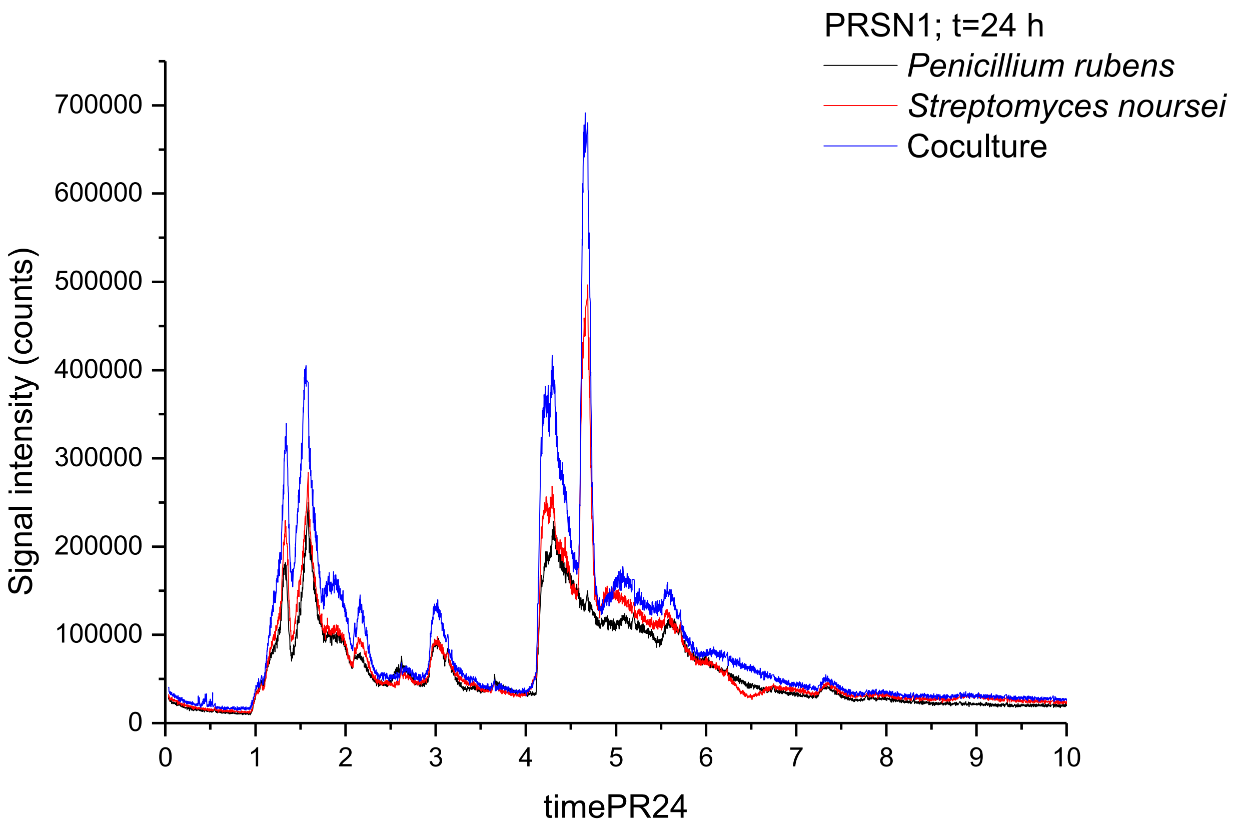

Figure S2. Alignments of total ion chromatograms (TICs) recorded in the PRSN1 experiment at t=24 h for *P. rubens* monoculture (black line), *S. noursei* monoculture (red line), and the "*P. rubens* vs. *S. noursei*" coculture (blue line).

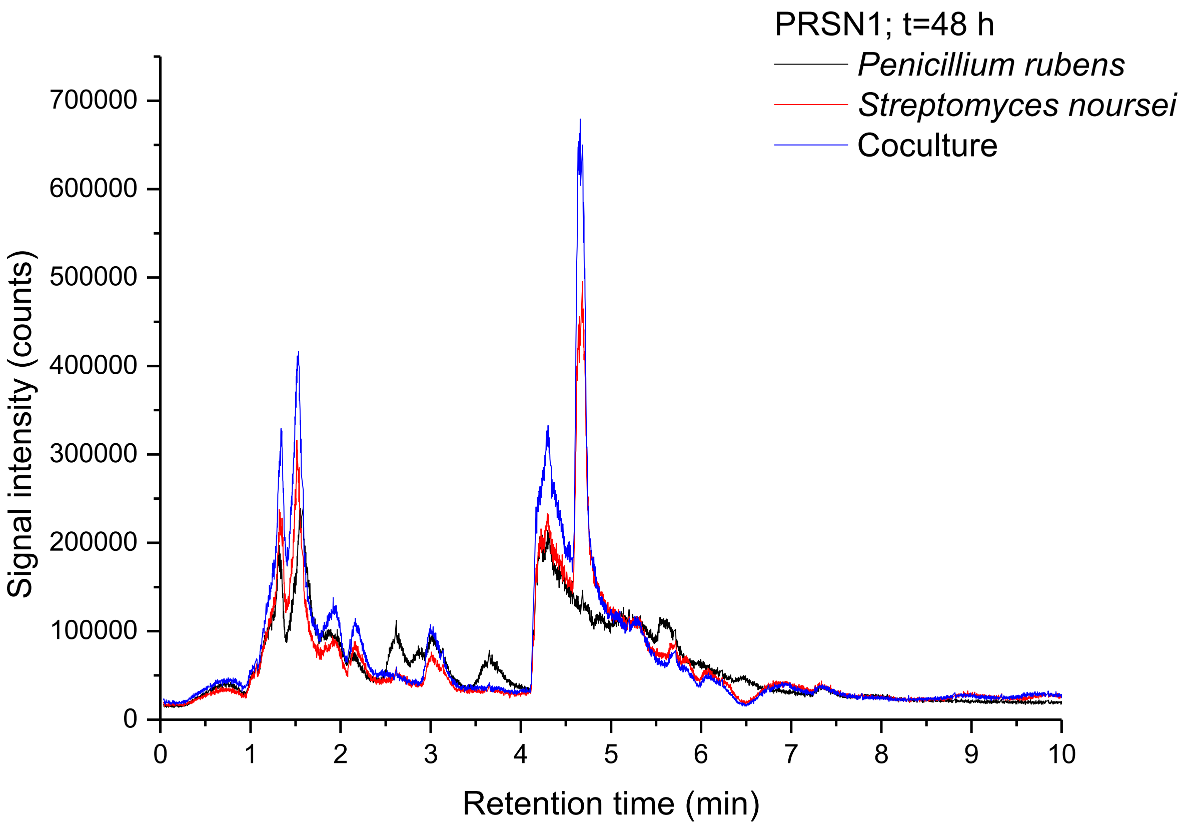

Figure S3. Alignments of total ion chromatograms (TICs) recorded in the PRSN1 experiment at t=48 h for *P. rubens* monoculture (black line), *S. noursei* monoculture (red line), and the "*P. rubens* vs. *S. noursei*" coculture (blue line).

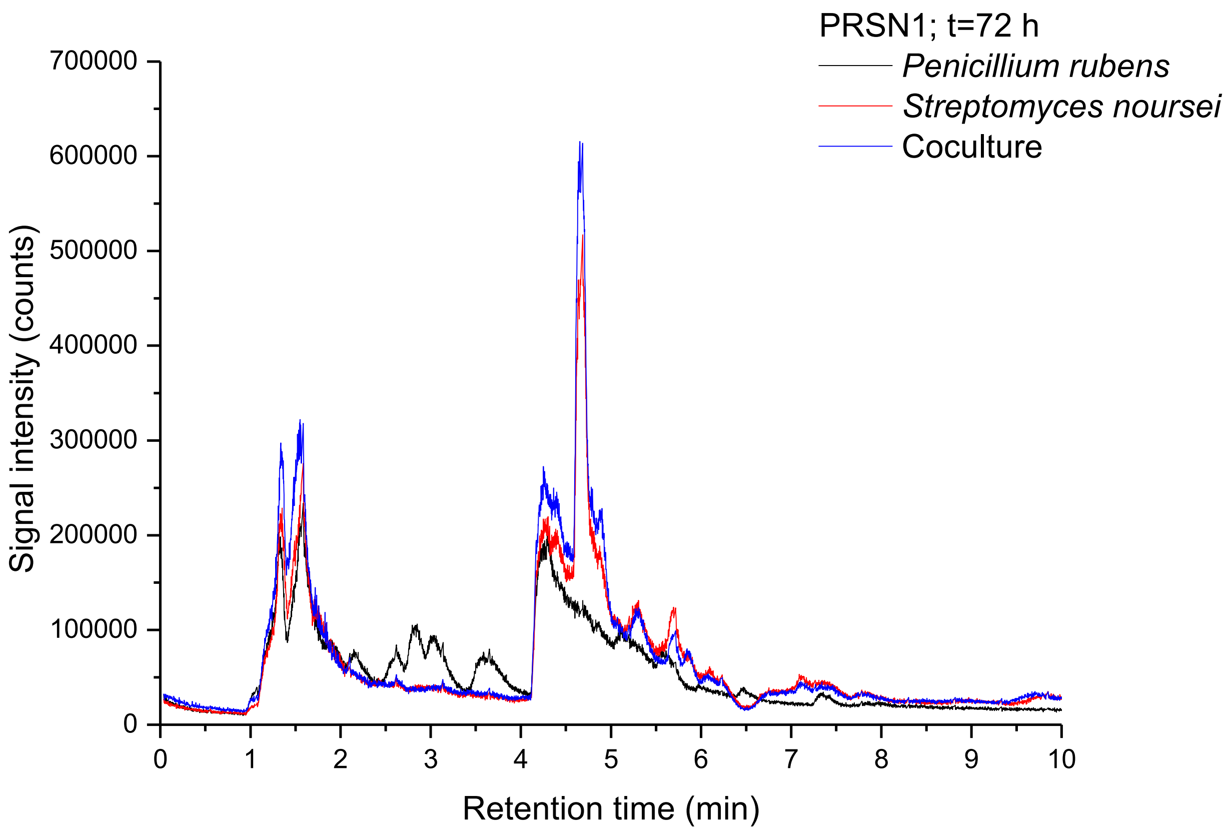

Figure S4. Alignments of total ion chromatograms (TICs) recorded in the PRSN1 experiment at t=72 h for *P. rubens* monoculture (black line), *S. noursei* monoculture (red line), and the "*P. rubens* vs. *S. noursei*" coculture (blue line).

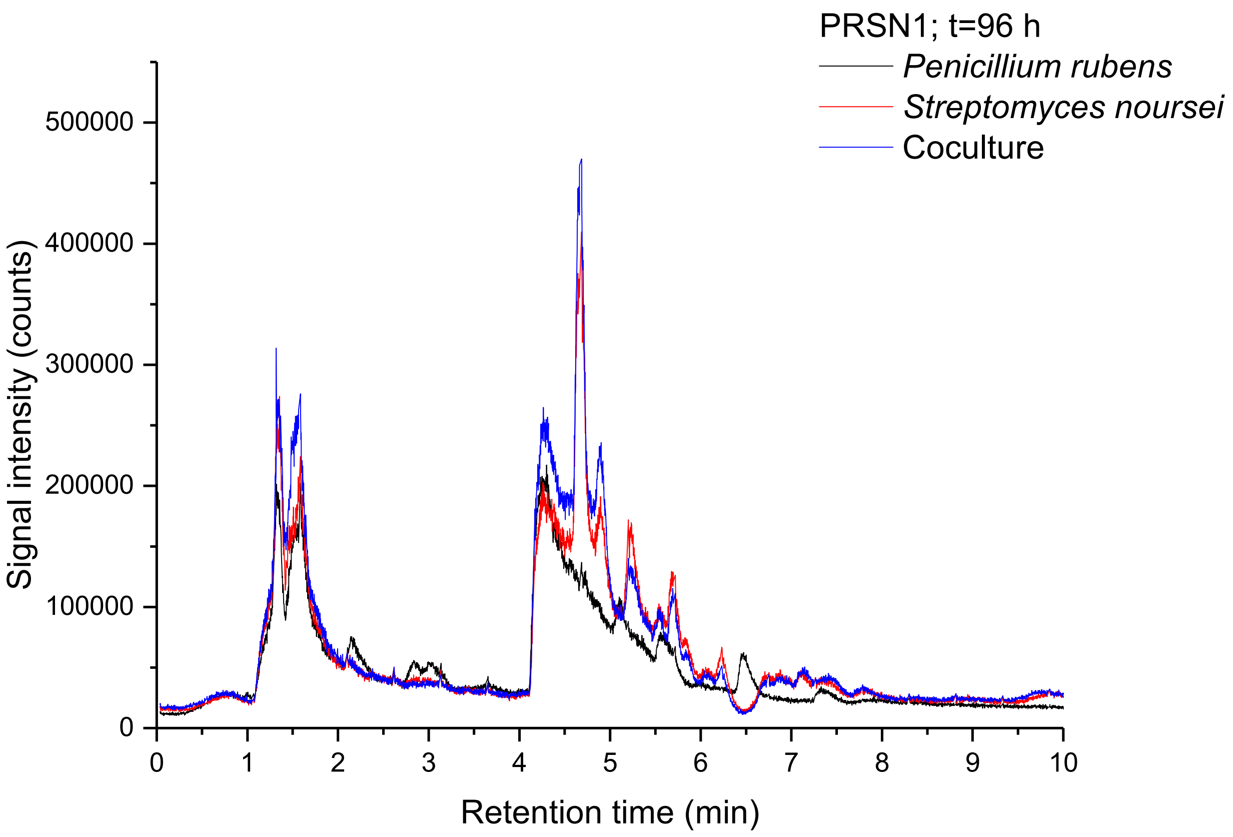

Figure S5. Alignments of total ion chromatograms (TICs) recorded in the PRSN1 experiment at t=96 h for *P. rubens* monoculture (black line), *S. noursei* monoculture (red line), and the "*P. rubens* vs. *S. noursei*" coculture (blue line).

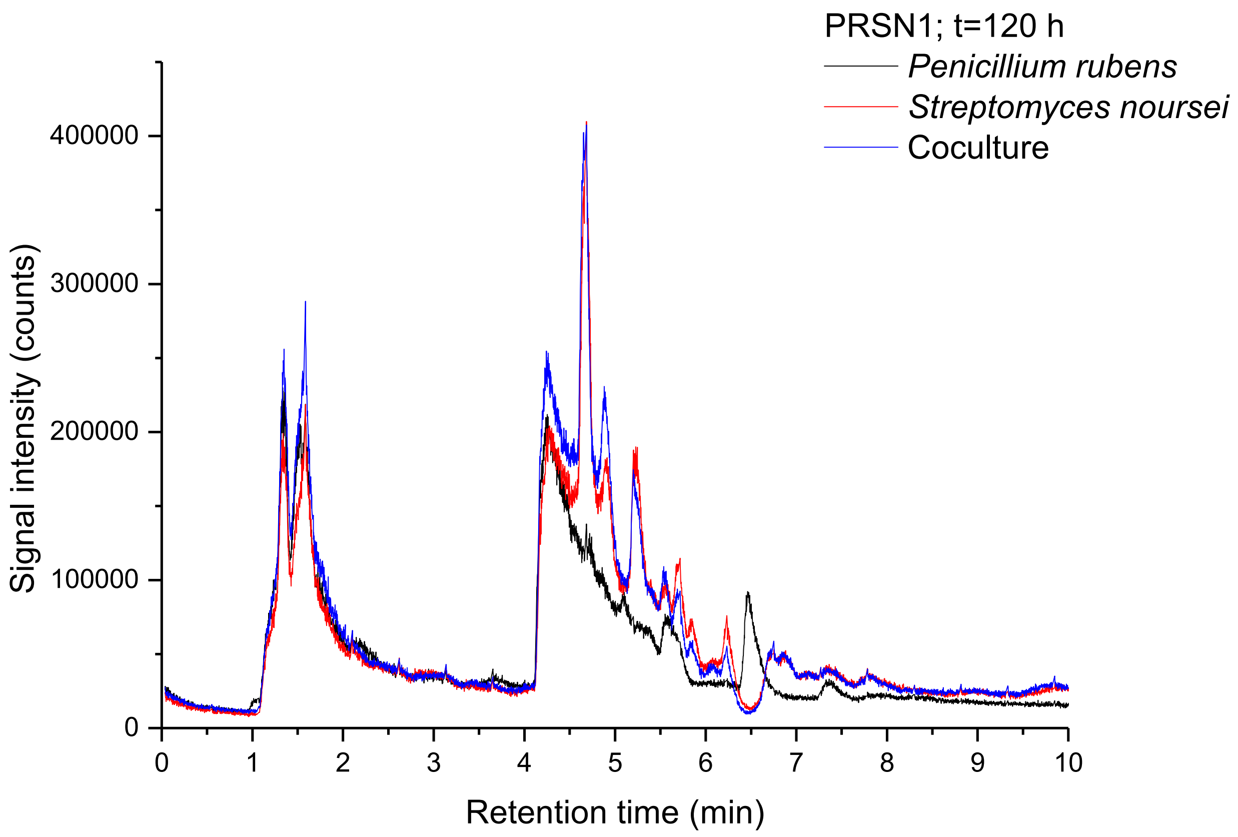

Figure S6. Alignments of total ion chromatograms (TICs) recorded in the PRSN1 experiment at t=120 h for *P. rubens* monoculture (black line), *S. noursei* monoculture (red line), and the "*P. rubens* vs. *S. noursei*" coculture (blue line).

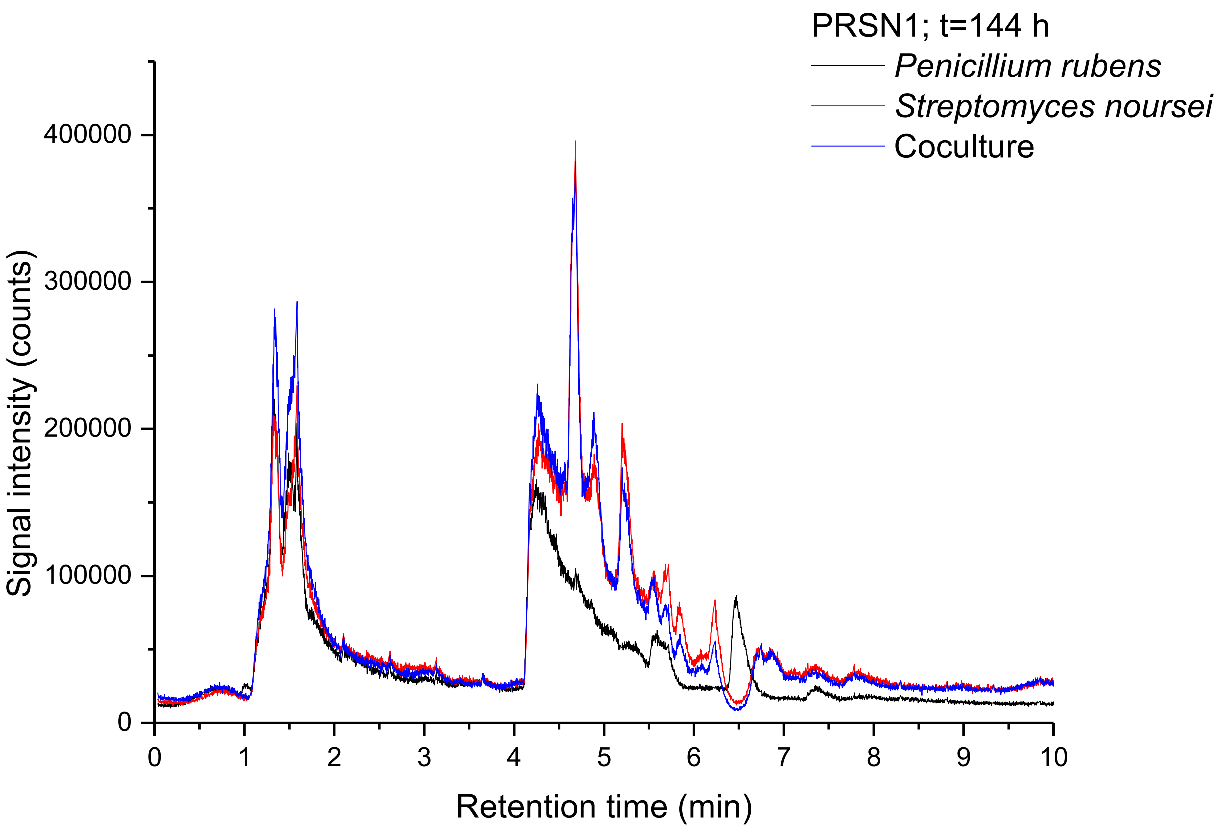

Figure S7. Alignments of total ion chromatograms (TICs) recorded in the PRSN1 experiment at t=144 h for *P. rubens* monoculture (black line), *S. noursei* monoculture (red line), and the "*P. rubens* vs. *S. noursei*" coculture (blue line).

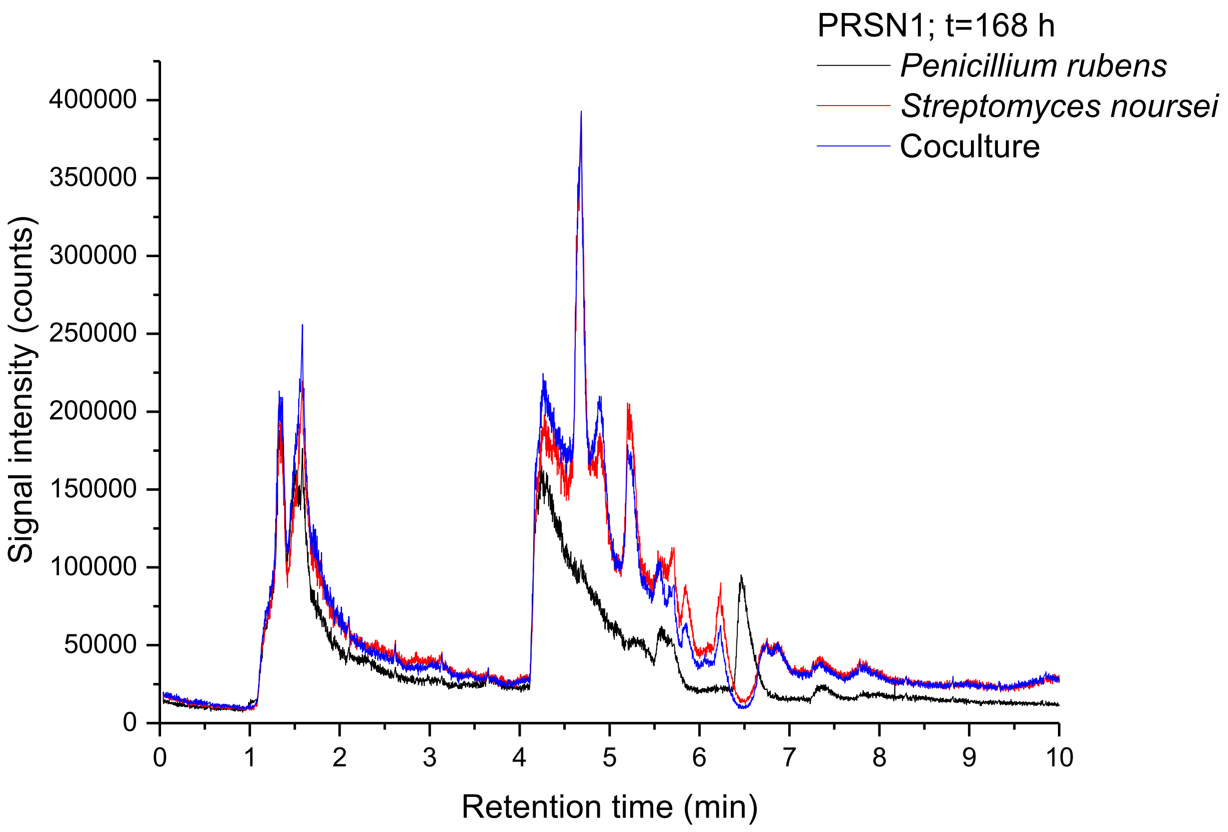

Figure S8. Alignments of total ion chromatograms (TICs) recorded in the PRSN1 experiment at t=168 h for *P. rubens* monoculture (black line), *S. noursei* monoculture (red line), and the "*P. rubens* vs. *S. noursei*" coculture (blue line).

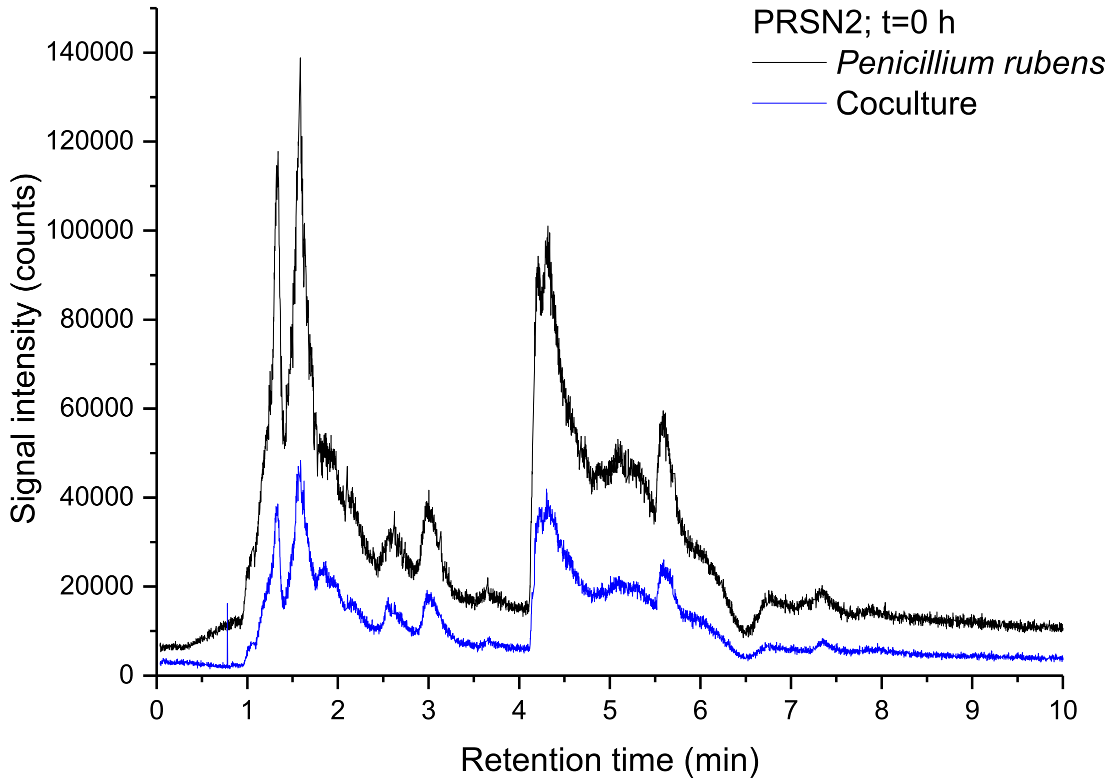

Figure S9. Alignments of total ion chromatograms (TICs) recorded in the PRSN2 experiment at t=0 h for *P. rubens* monoculture (black line) and the "*P. rubens* vs. *S. noursei*" coculture (blue line).

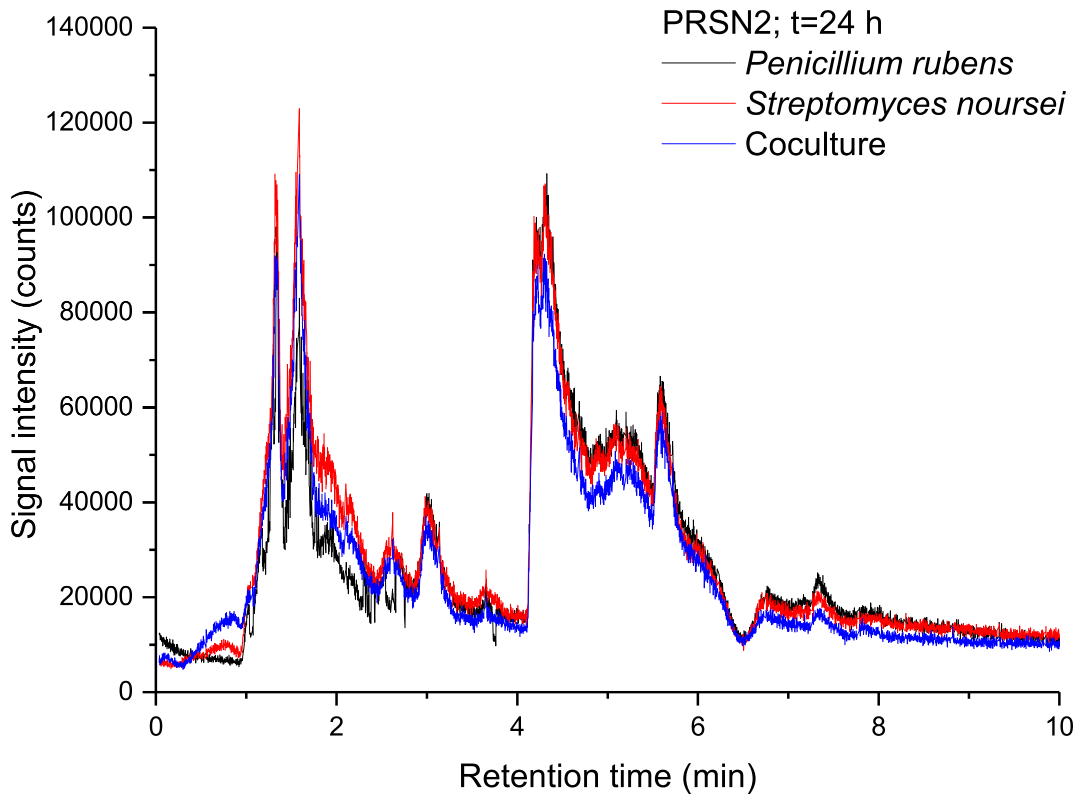

Figure S10. Alignments of total ion chromatograms (TICs) recorded in the PRSN2 experiment at t=24 h for *P. rubens* monoculture (black line), *S. noursei* monoculture (red line), and the "*P. rubens* vs. *S. noursei*" coculture (blue line).

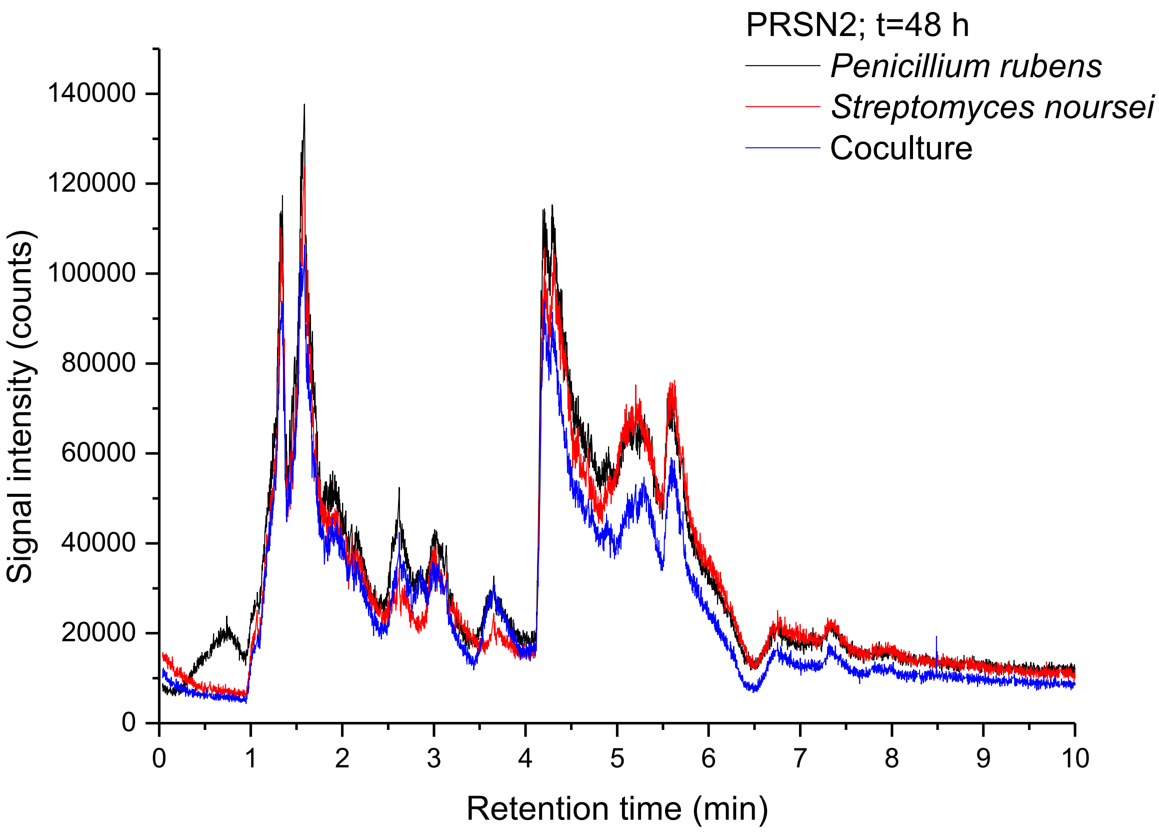

Figure S11. Alignments of total ion chromatograms (TICs) recorded in the PRSN2 experiment at t=48 h for *P. rubens* monoculture (black line), *S. noursei* monoculture (red line), and the "*P. rubens* vs. *S. noursei*" coculture (blue line).

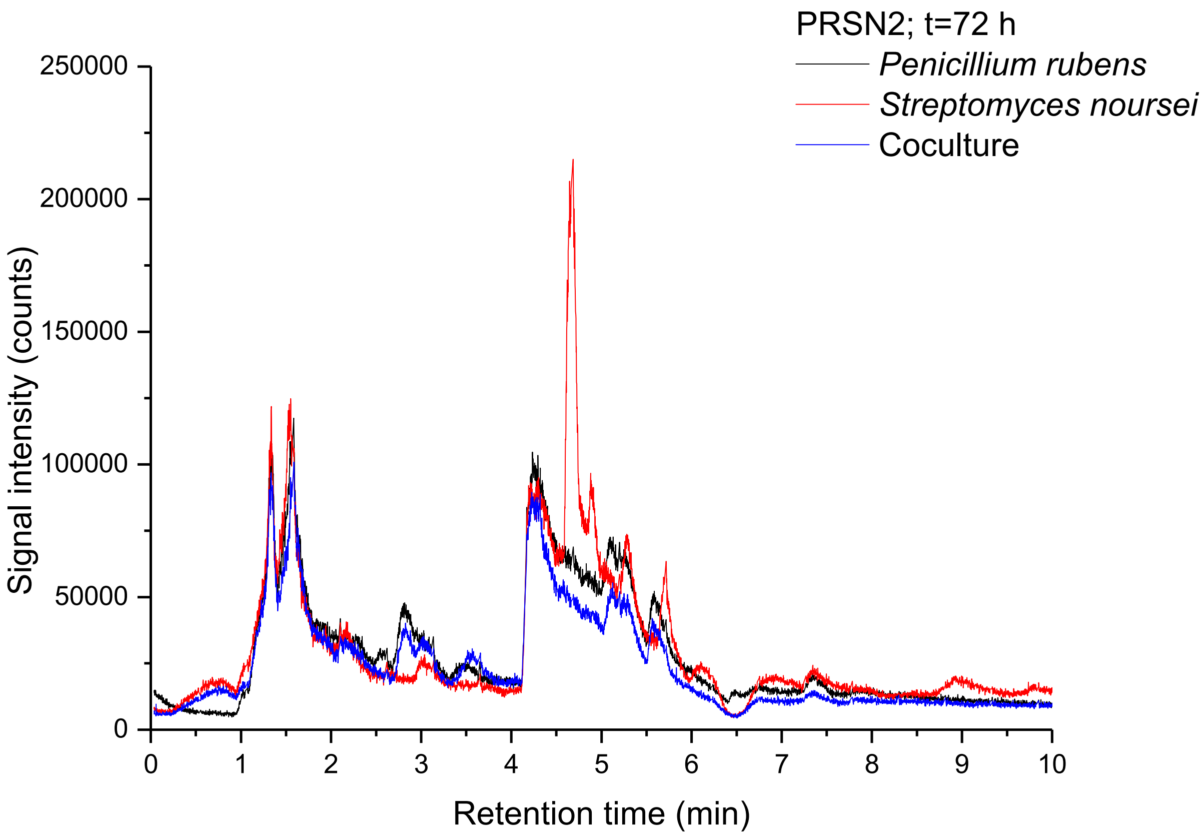

Figure S12. Alignments of total ion chromatograms (TICs) recorded in the PRSN2 experiment at t=72 h for *P. rubens* monoculture (black line), *S. noursei* monoculture (red line), and the "*P. rubens* vs. *S. noursei*" coculture (blue line).

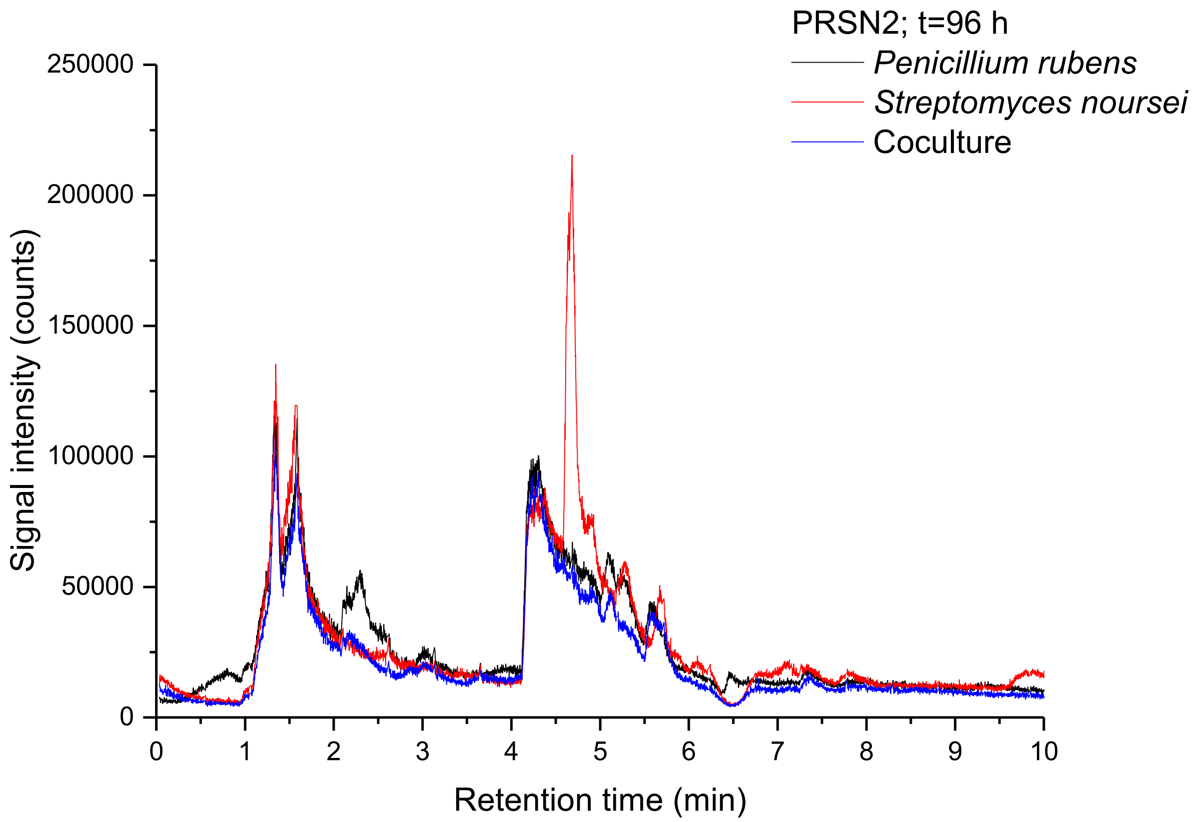

Figure S13. Alignments of total ion chromatograms (TICs) recorded in the PRSN2 experiment at t=96 h for *P. rubens* monoculture (black line), *S. noursei* monoculture (red line), and the "*P. rubens* vs. *S. noursei*" coculture (blue line).

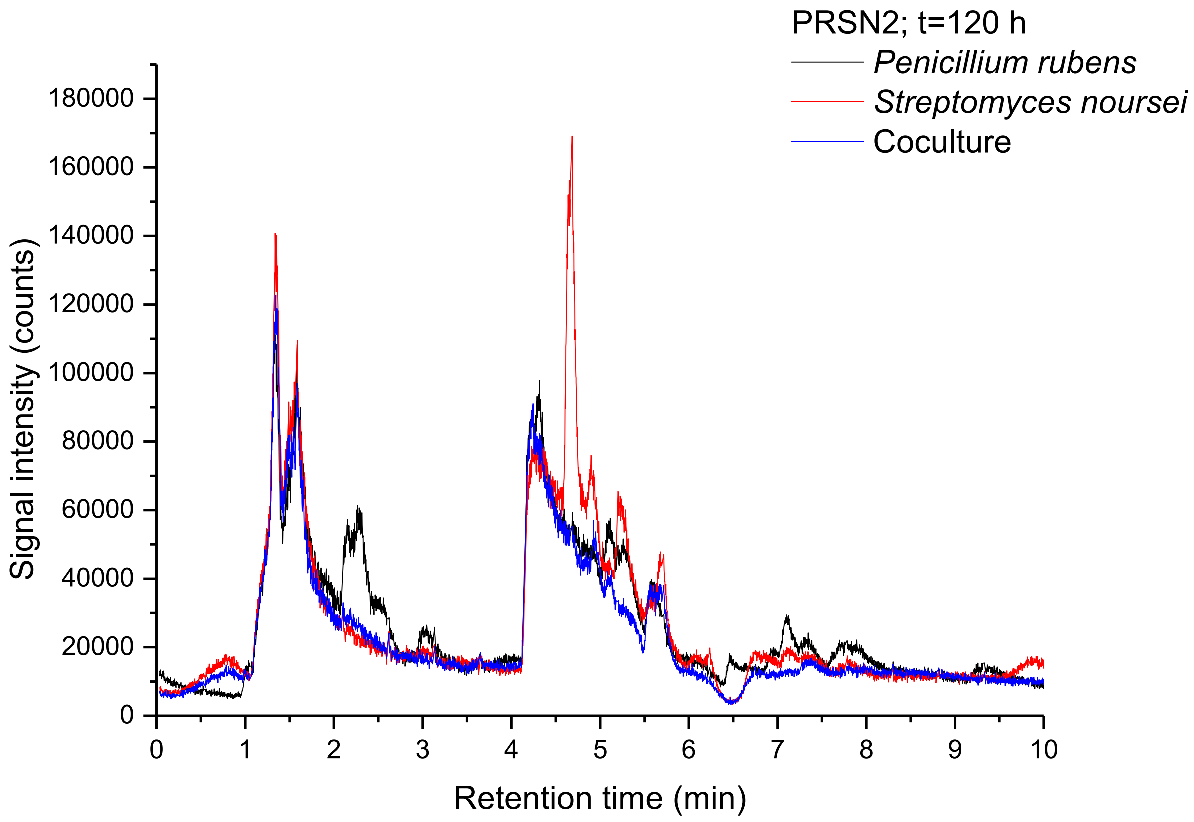

Figure S14. Alignments of total ion chromatograms (TICs) recorded in the PRSN2 experiment at t=120 h for *P. rubens* monoculture (black line), *S. noursei* monoculture (red line), and the "*P. rubens* vs. *S. noursei*" coculture (blue line).

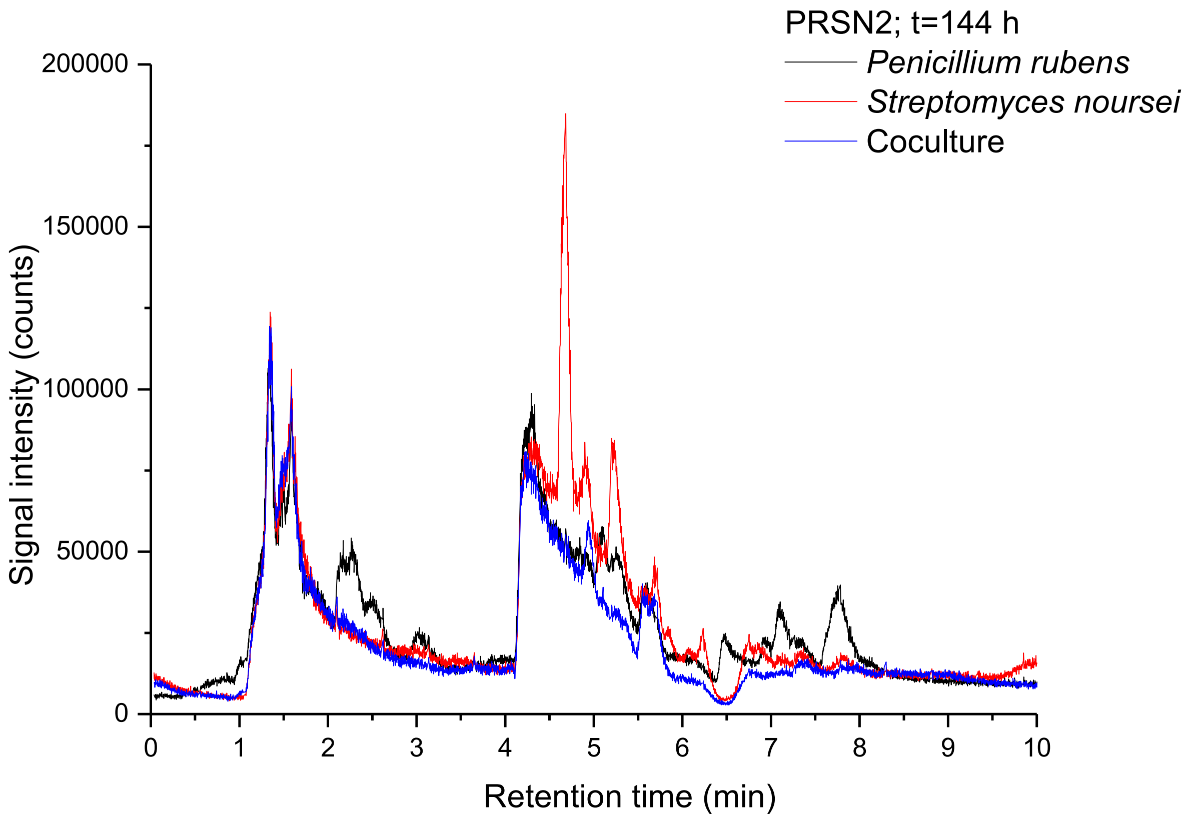

Figure S15. Alignments of total ion chromatograms (TICs) recorded in the PRSN2 experiment at t=144 h for *P. rubens* monoculture (black line), *S. noursei* monoculture (red line), and the "*P. rubens* vs. *S. noursei*" coculture (blue line).

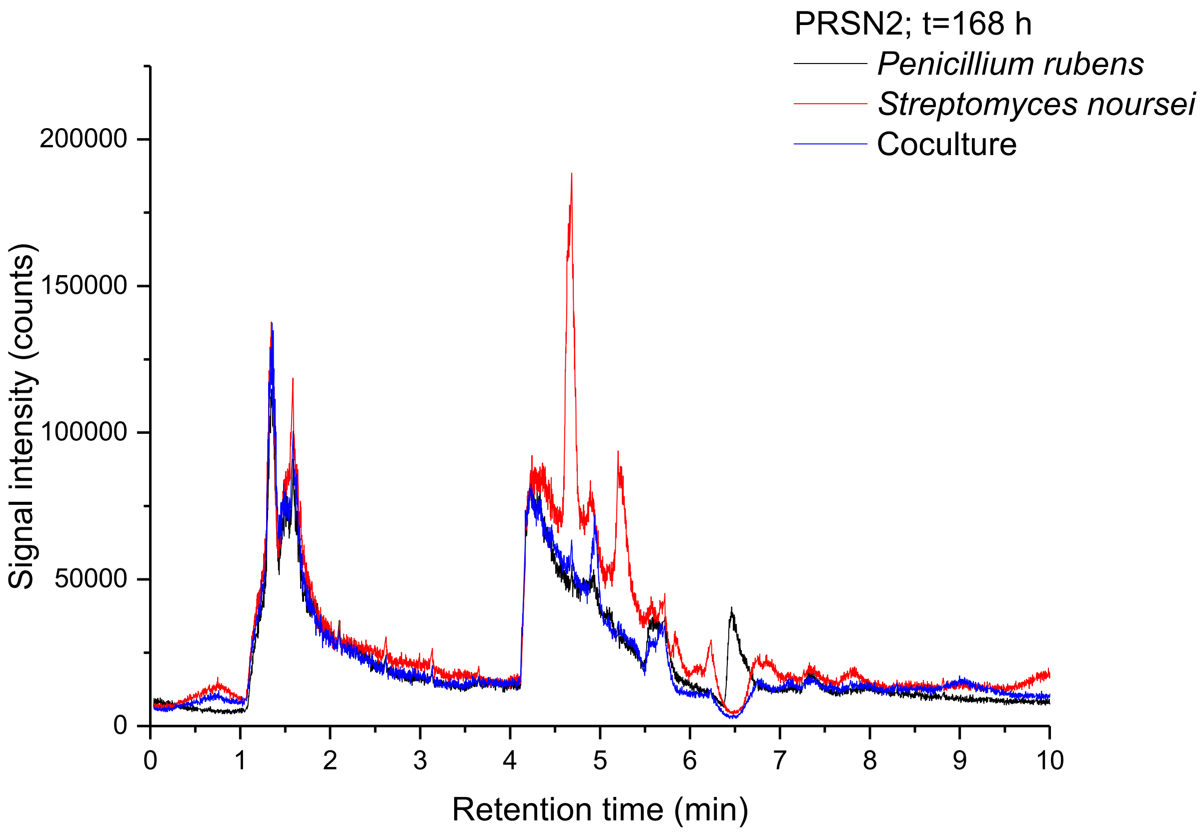

Figure S16. Alignments of total ion chromatograms (TICs) recorded in the PRSN2 experiment at t=168 h for *P. rubens* monoculture (black line), *S. noursei* monoculture (red line), and the "*P. rubens* vs. *S. noursei*" coculture (blue line).

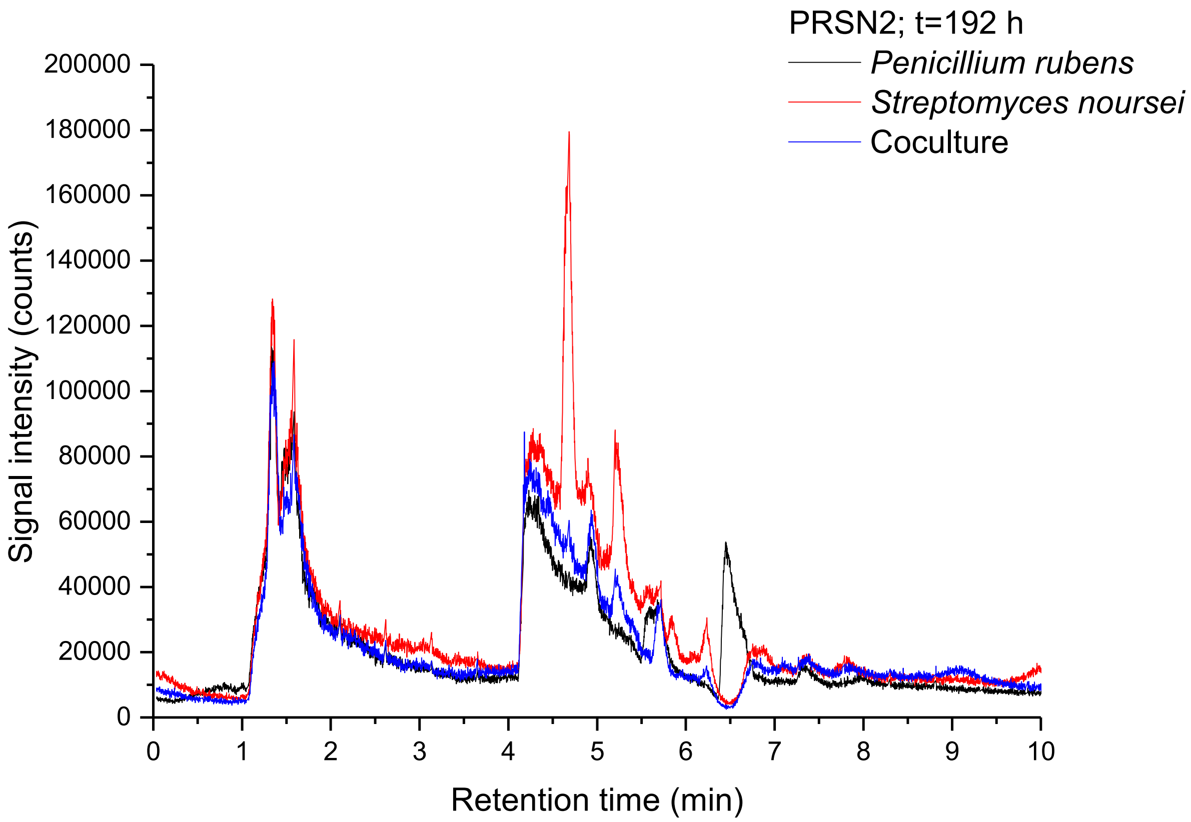

Figure S17. Alignments of total ion chromatograms (TICs) recorded in the PRSN2 experiment at t=192 h for *P. rubens* monoculture (black line), *S. noursei* monoculture (red line), and the "*P. rubens* vs. *S. noursei*" coculture (blue line).

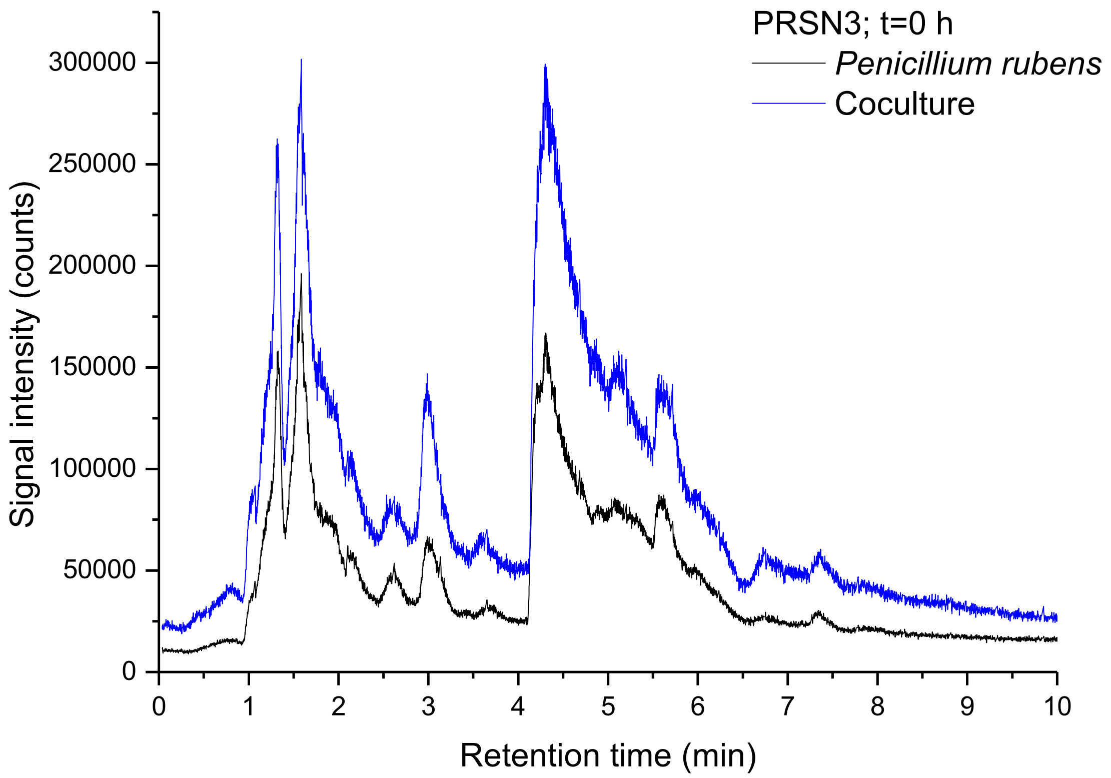

Figure S18. Alignments of total ion chromatograms (TICs) recorded in the PRSN3 experiment at t=0 h for *P. rubens* monoculture (black line) and the "*P. rubens* vs. *S. noursei*" coculture (blue line).

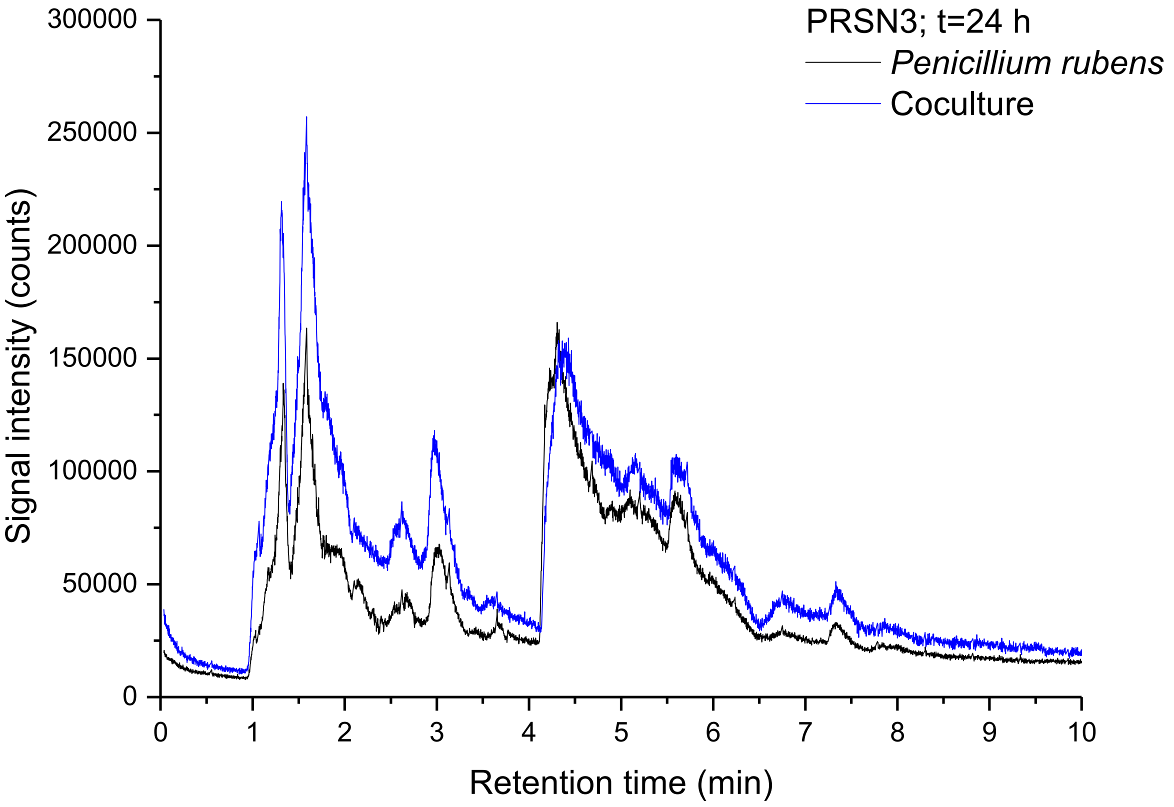

Figure S19. Alignments of total ion chromatograms (TICs) recorded in the PRSN3 experiment at t=24 h for *P. rubens* monoculture (black line) and the "*P. rubens* vs. *S. noursei*" coculture (blue line).

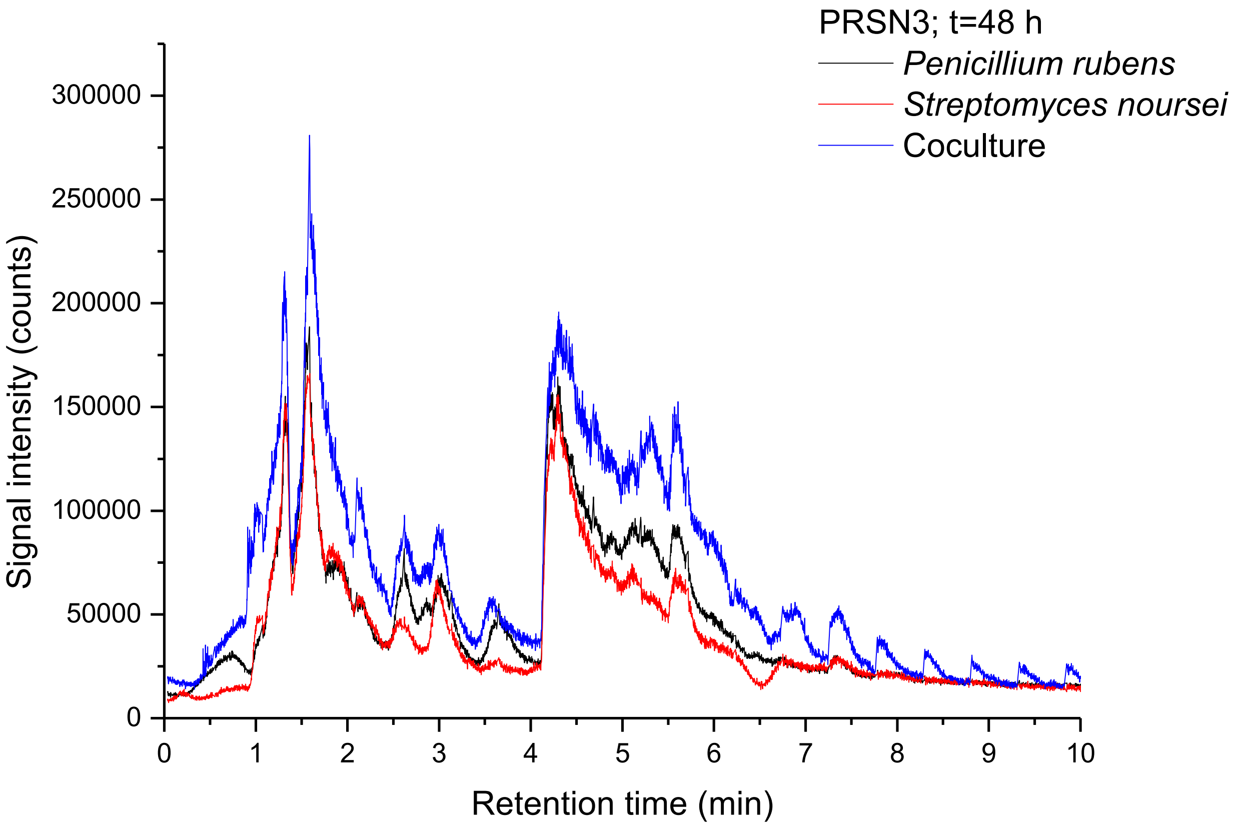

Figure S20. Alignments of total ion chromatograms (TICs) recorded in the PRSN3 experiment at t=48 h for *P. rubens* monoculture (black line), *S. noursei* monoculture (red line), and the "*P. rubens* vs. *S. noursei*" coculture (blue line).

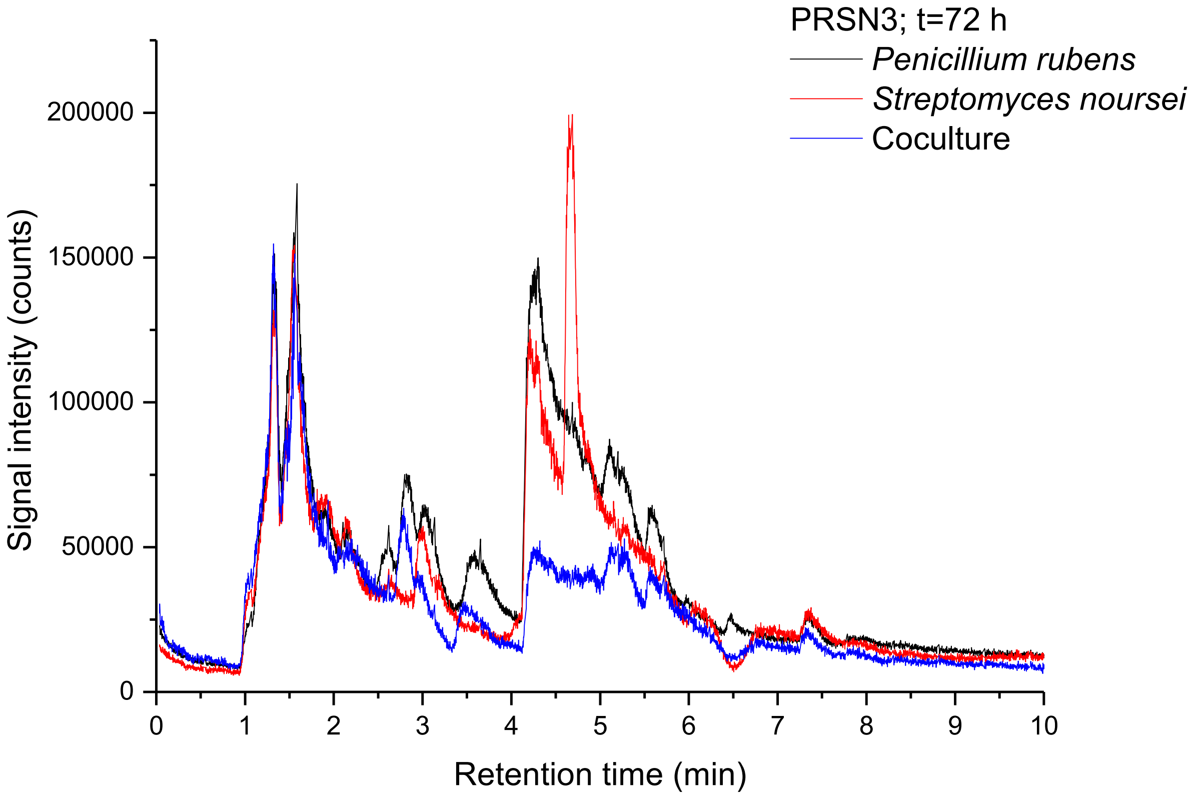

Figure S21. Alignments of total ion chromatograms (TICs) recorded in the PRSN3 experiment at t=72 h for *P. rubens* monoculture (black line), *S. noursei* monoculture (red line), and the "*P. rubens* vs. *S. noursei*" coculture (blue line).

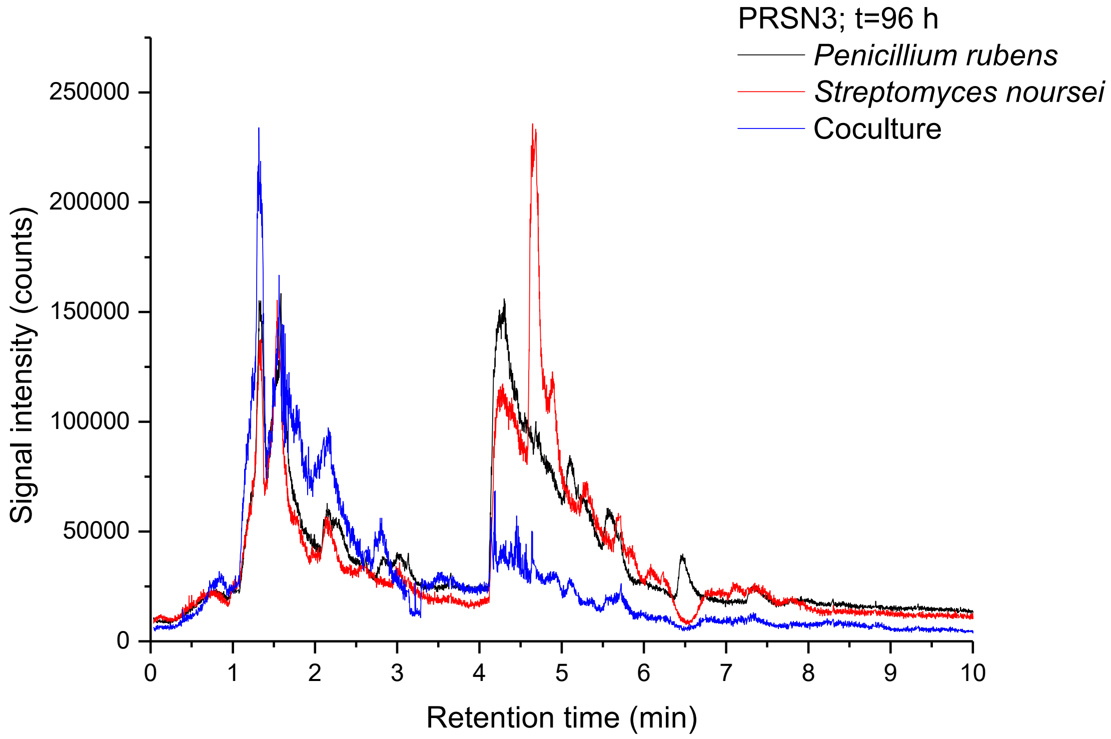

Figure S22. Alignments of total ion chromatograms (TICs) recorded in the PRSN3 experiment at t=96 h for *P. rubens* monoculture (black line), *S. noursei* monoculture (red line), and the "*P. rubens* vs. *S. noursei*" coculture (blue line).

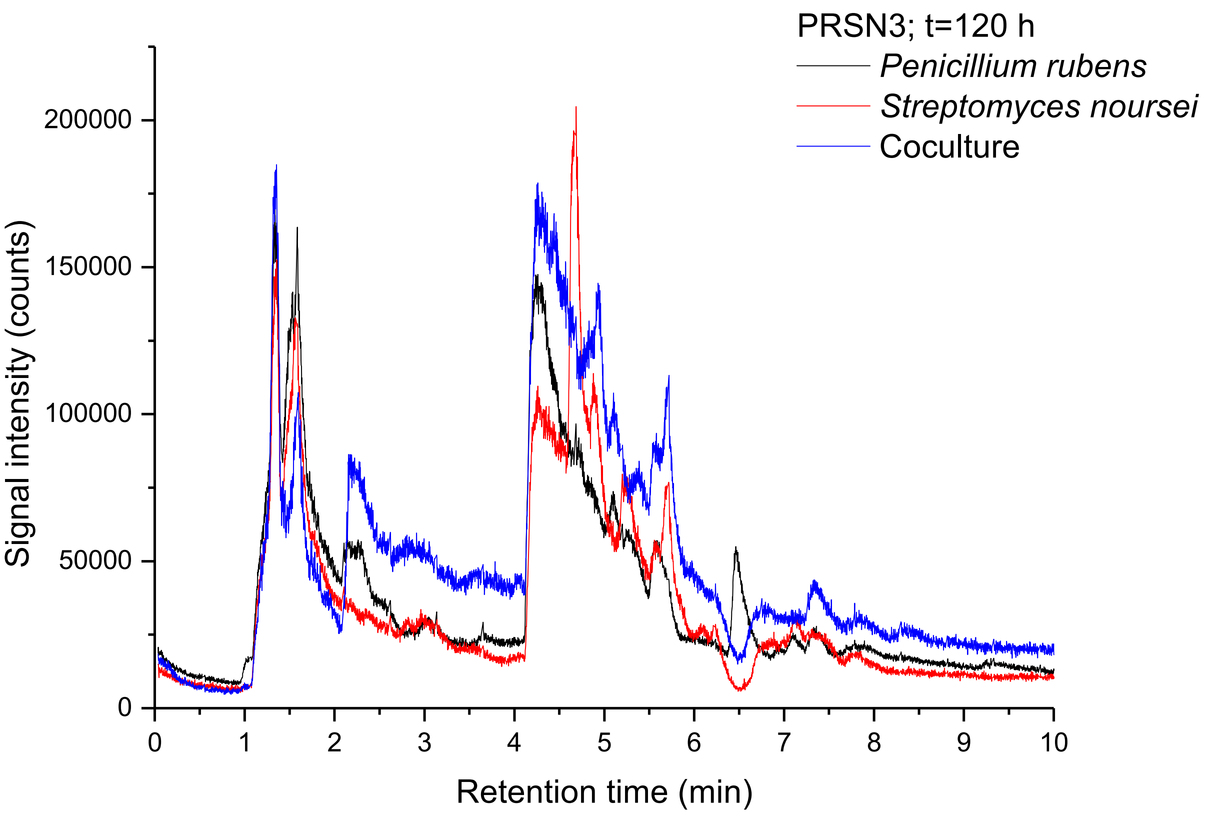

Figure S23. Alignments of total ion chromatograms (TICs) recorded in the PRSN3 experiment at t=120 h for *P. rubens* monoculture (black line), *S. noursei* monoculture (red line), and the "*P. rubens* vs. *S. noursei*" coculture (blue line).

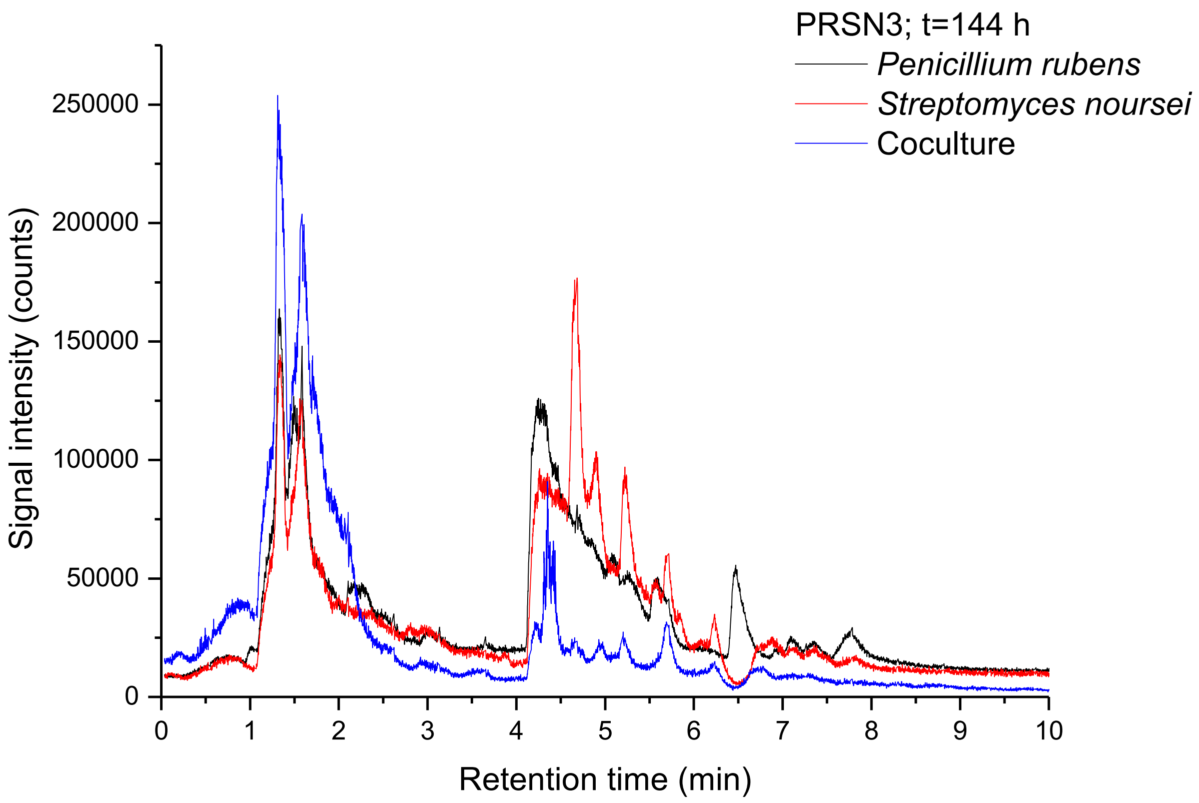

Figure S24. Alignments of total ion chromatograms (TICs) recorded in the PRSN3 experiment at t=144 h for *P. rubens* monoculture (black line), *S. noursei* monoculture (red line), and the "*P. rubens* vs. *S. noursei*" coculture (blue line).

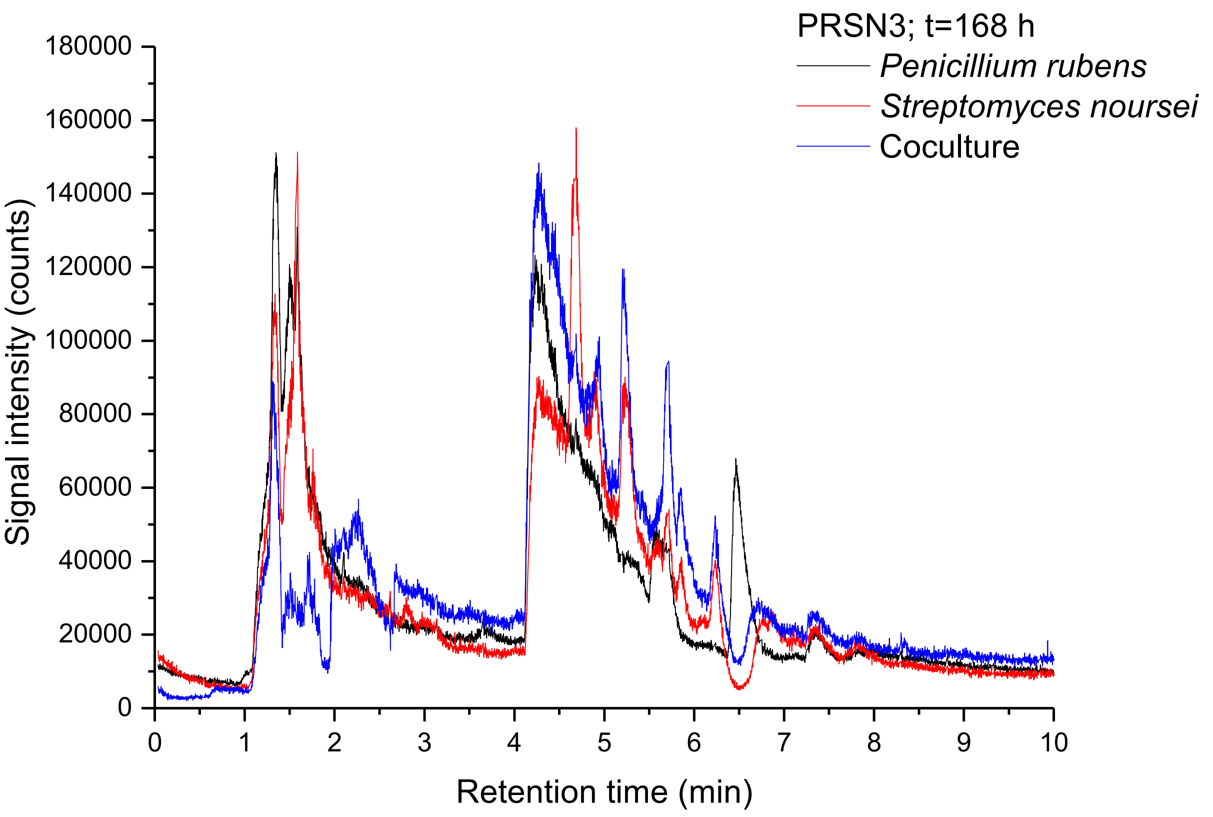

Figure S25. Alignments of total ion chromatograms (TICs) recorded in the PRSN3 experiment at t=168 h for *P. rubens* monoculture (black line), *S. noursei* monoculture (red line), and the "*P. rubens* vs. *S. noursei*" coculture (blue line).

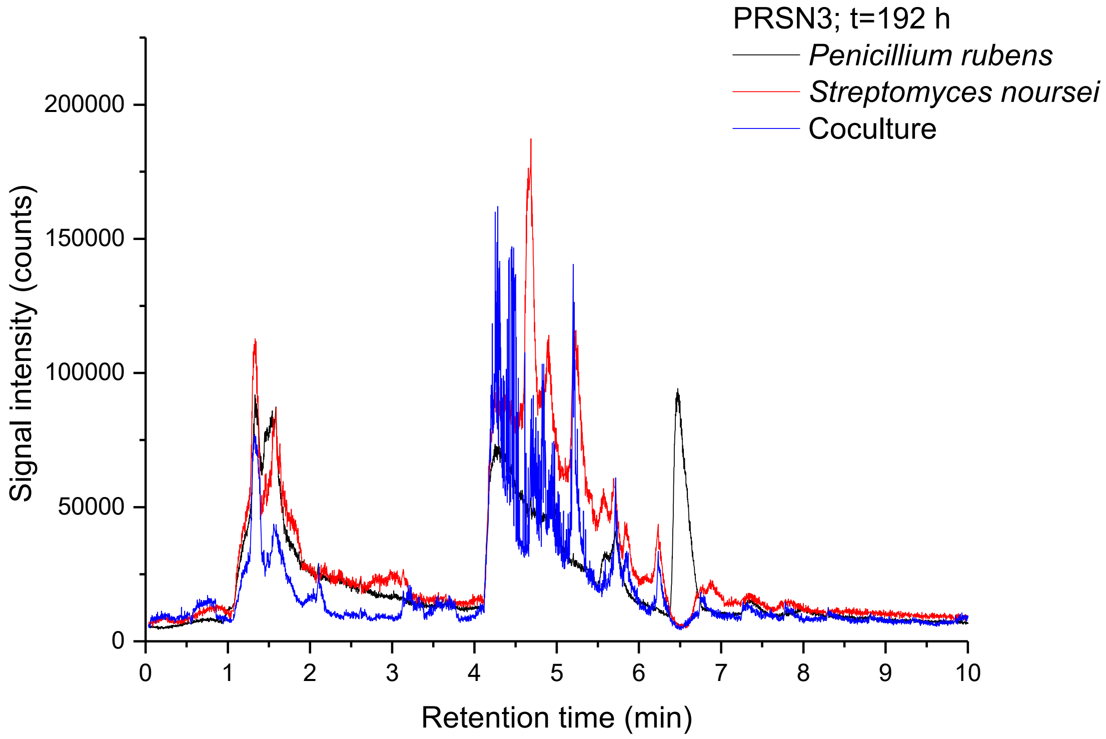

Figure S26. Alignments of total ion chromatograms (TICs) recorded in the PRSN3 experiment at t=192 h for *P. rubens* monoculture (black line), *S. noursei* monoculture (red line), and the "*P. rubens* vs. *S. noursei*" coculture (blue line).

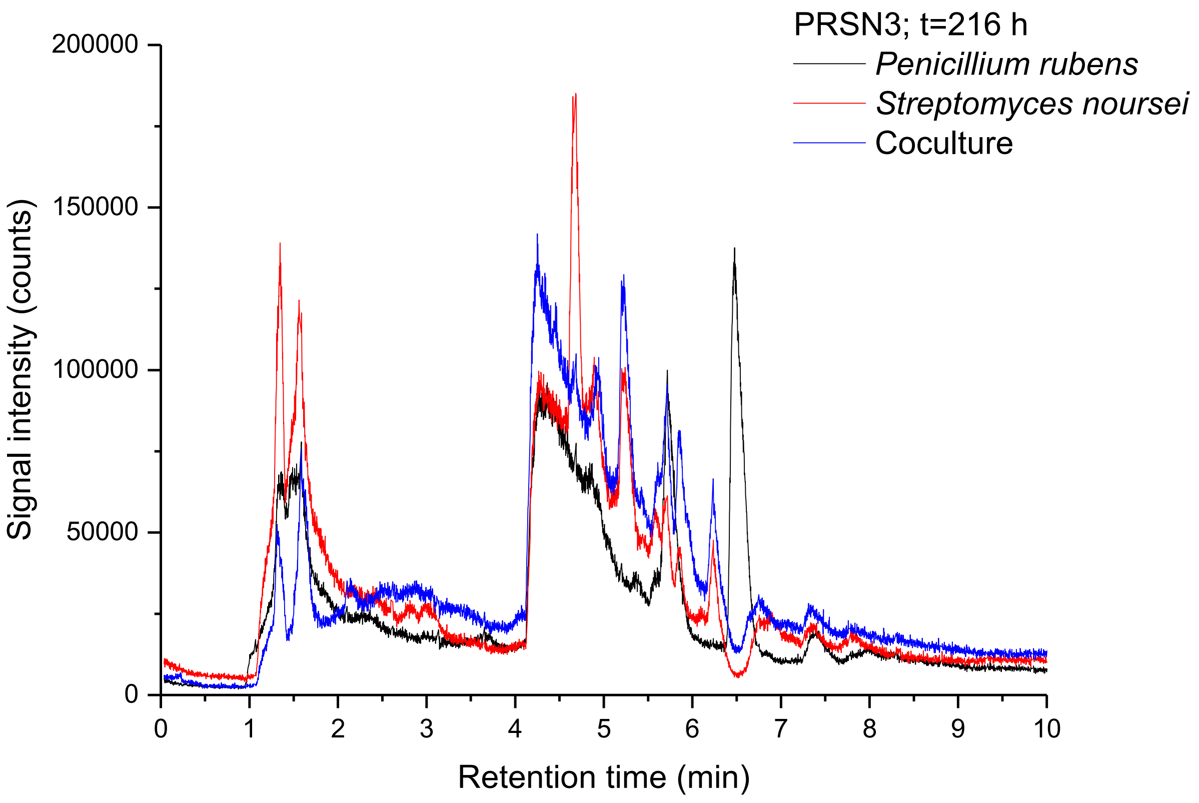

Figure S27. Alignments of total ion chromatograms (TICs) recorded in the PRSN3 experiment at t=216 h for *P. rubens* monoculture (black line), *S. noursei* monoculture (red line), and the "*P. rubens* vs. *S. noursei*" coculture (blue line).

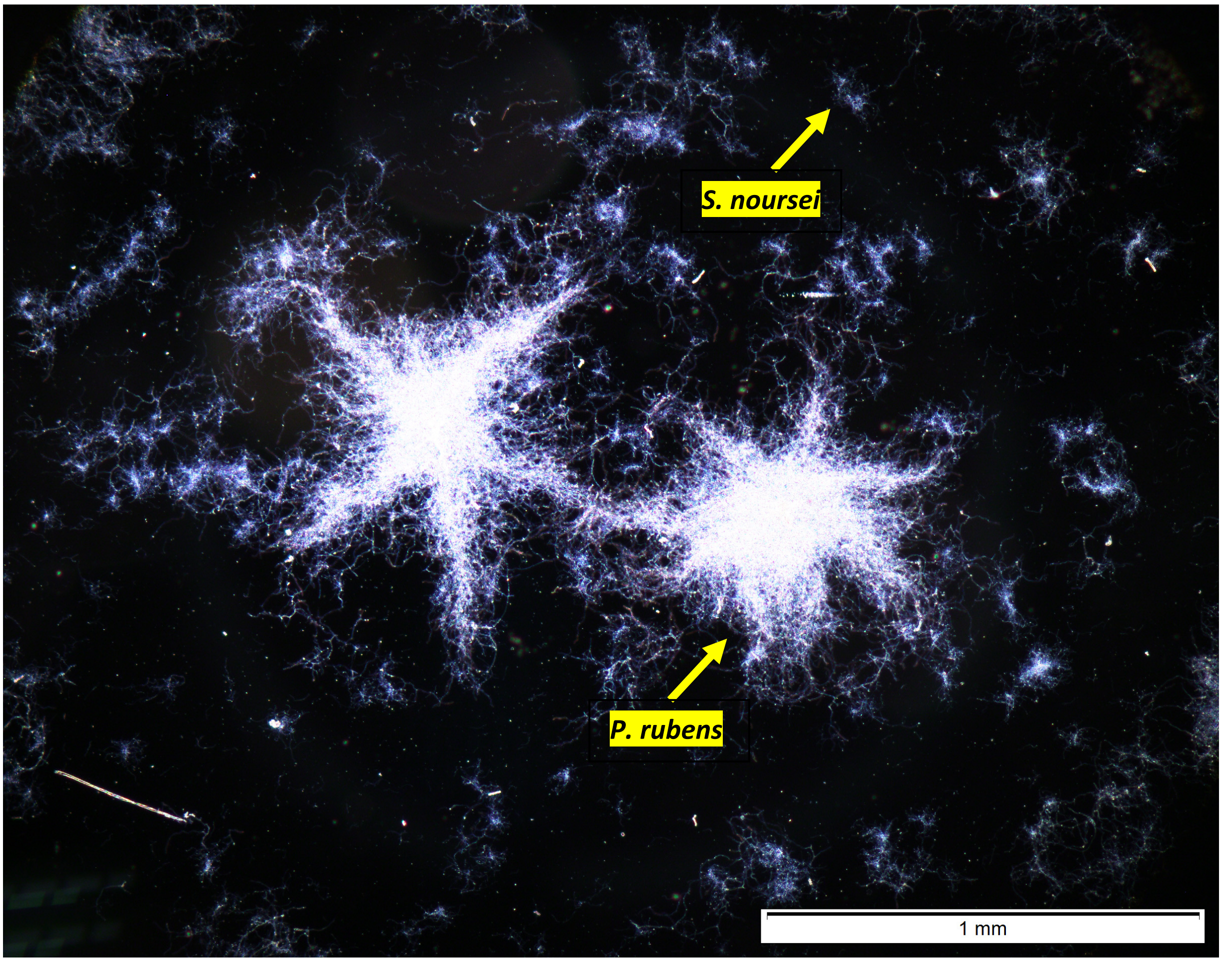

Figure S28. Microscopic image of the PRSN3 co-culture taken at 144 h of the process.

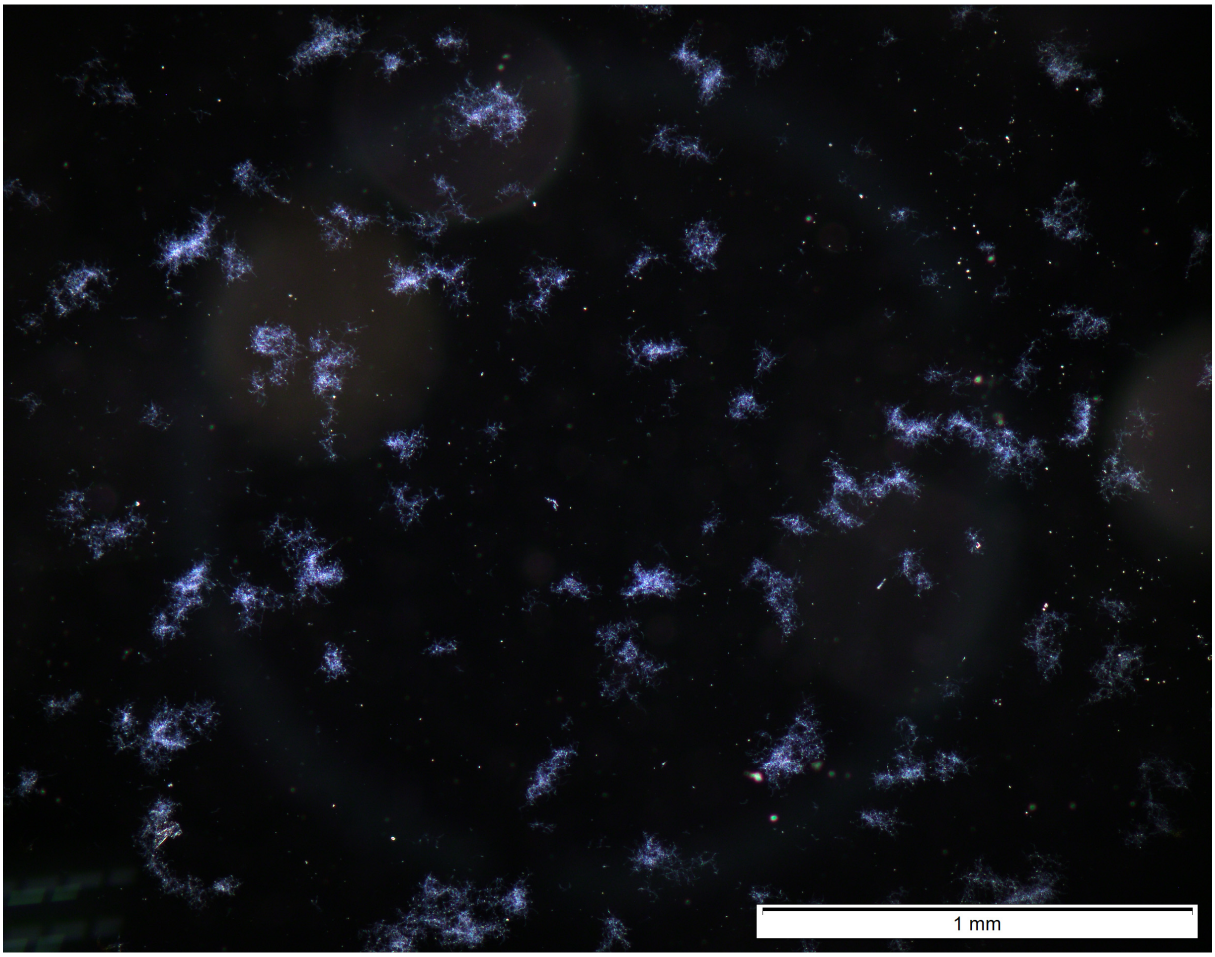

Figure S29. Microscopic image of the *S. noursei* monoculture taken at 144 h of the PRSN3 process.

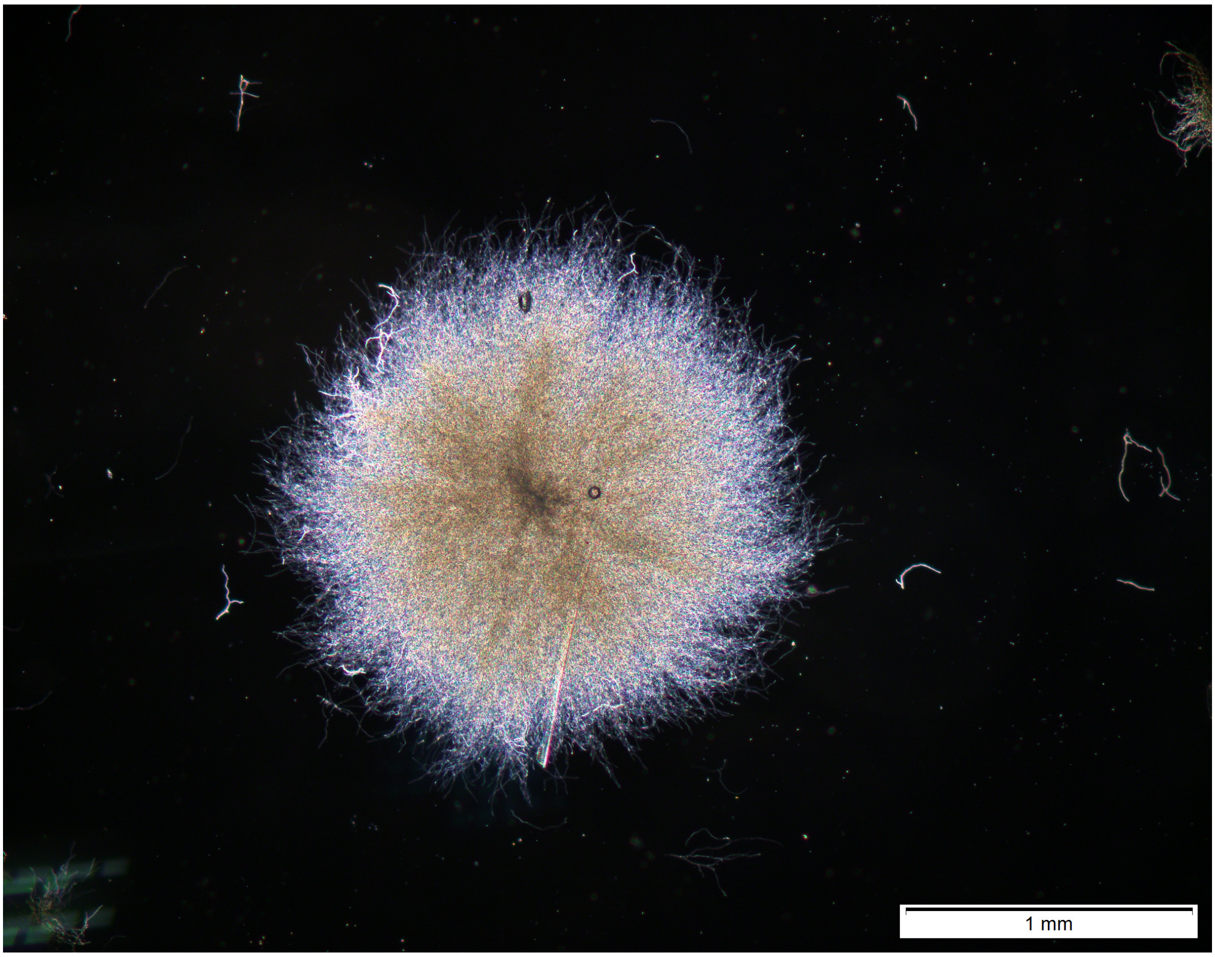

Figure S30. Microscopic image of the *P. rubens* monoculture taken at 144 h of the PRSN3 process.
